# * In vitro* modulation of the human gut bacterial community by crude polysaccharides from cultivated mushrooms *Lentinus polychrous* and *Lentinus squarrosulus*

**DOI:** 10.7717/peerj.21348

**Published:** 2026-06-10

**Authors:** Marutpong Panya, Phairo Saenwang, Pawana Panomket, Uthai Unphim

**Affiliations:** 1 Research Group for Biomedical Research and Innovative Development (RG-BRID), College of Medicine and Public Health, Ubon Ratchathani University, Warinchamrap, Ubon Ratchathani, Thailand; 2 Faculty of Management Science, Ubon Ratchathani University, Warinchamrap, Ubon Ratchathani, Thailand

**Keywords:** *Lentinus polychrous*, *Lentinus squarrosulus*, Polysaccharide, Prebiotic, Human fecal bacterial community, *In vitro* fecal fermentation

## Abstract

**Background:**

Edible mushroom polysaccharides are recognized as prebiotics that influence gut microbiota composition and host health. However, the effects of the polysaccharides from *Lentinus squarrosulus* and *Lentinus polychrous* on the human gut bacterial community remain poorly understood. To address this gap, the present study evaluated the effects of crude polysaccharide extracts from cultivated mushrooms on the human fecal bacterial community using an *in vitro* batch fermentation model.

**Methods:**

Species identification of the two mushroom isolates (PB1 and LP1) was performed using molecular techniques. Cultivated mushrooms served as a source of polysaccharides, which were extracted using a combination of sonication and hot water. The composition of crude polysaccharides, including total carbohydrates, reducing sugars, proteins, and phenolic compounds, was analyzed. These extracts were subjected to simulated gastrointestinal digestion and used as substrates in batch fermentation inoculated with human fecal slurry. The bacterial communities in the treatment groups were identified and analyzed using 16S rRNA gene amplicon sequencing.

**Results:**

PB1 and LP1 were identified as *L. squarrosulus* PB1 and *L. polychrous* LP1, respectively. Composition analysis revealed that the crude polysaccharides LS and LP derived from *L. squarrosulus* PB1 and *L. polychrous* LP1, respectively, were primarily composed of polysaccharides with low levels of protein, reducing sugars, and phenolic compounds. After the simulated gastrointestinal digestion, LS and LP exhibited high resistance, suggesting that they may reach the colon for microbial fermentation. Fermentation of LS and LP modulated the gut microbiota by increasing the abundance of beneficial genera such as *Bacteroides* and *Parabacteroides.* A notable increase in the relative abundance of *Fusobacterium* was observed. However, its functional role requires further investigation. Additionally, fermentation with LS or LP reduced both Firmicutes/Bacteroidota and Blautia/Bacteroides ratios. Fermentation also resulted in a decrease in pH.

**Conclusions:**

Crude polysaccharides from cultivated *L. squarrosulus* PB1 and *L. polychrous* LP1 can modulate the human fecal bacterial community by enriching beneficial bacteria, highlighting their potential to improve gut health. Further research is needed to elucidate how the specific characteristics of these polysaccharides, particularly their structure, influence the gut microbiota and assess their implications for human health.

## Introduction

The gut bacterial community, defined as the composition and abundance of all bacterial taxa in the human gastrointestinal tract, has been the most intensively investigated component of the human microbiome because it comprises the predominant microorganisms inhabiting this system (Sender, Fuchs & Milo, 2016). Imbalances in this community, known as dysbiosis, have been associated with the development of several diseases, including asthma, obesity, cardiovascular disease, autism, and cancer (Fujimura & Lynch, 2015; Long et al., 2023; Peralta-Marzal et al., 2024; Turnbaugh et al., 2006; Zuo et al., 2019). This has led to the development of novel preventive and therapeutic strategies. Recent studies have highlighted the therapeutic potential of prebiotics in disease management through restoration of the gut microbiome, particularly by rebalancing the bacterial community (Bevilacqua et al., 2024).

Prebiotics are substrates that are selectively utilized by host microorganisms to provide health benefits (Gibson et al., 2017). Unlike other nutrients, prebiotics resist digestion in the human gastrointestinal tract and are fermented by gut microorganisms, particularly probiotics. This fermentation process yields short-chain fatty acids (SCFAs) such as acetic, butyric, and propionic acids, and other metabolites that support the health of vital organs, including the brain and heart (Davani-Davari et al., 2019; Rahman et al., 2022). Edible mushrooms have emerged as a significant source of prebiotic compounds, particularly polysaccharides such as β-glucans. *In vitro* studies have demonstrated that polysaccharides extracted from various wild mushrooms can stimulate the growth of probiotic strains such as *Lactobacillus acidophilus* and *Lactobacillus rhamnosus* (Nowak et al., 2018). Furthermore, *in vitro* fecal fermentation studies have revealed that polysaccharides from mushrooms, including *Pleurotus eryngii*, *Tremella fuciformis*, *Agaricus bisporus*, *Hericium erinaceus*, and the dried fruiting body powder of *Lentinus squarrosulus,* can positively alter the gut microbiota community by increasing the population of beneficial bacteria, notably *Bifidobacterium* species (Ayimbila et al., 2022; Christodoulou et al., 2023; Duan et al., 2023; Priori et al., 2024; Zhu et al., 2024). These findings underscore the promising role of mushroom-derived polysaccharides in modulating gut bacterial communities.

In this study, we focused on two wild edible mushrooms, *L. squarrosulus* PB1 and *L. polychrous* LP1, identified using molecular techniques. These mushrooms were selected because they are abundant in Southeast Asia, including Thailand, and are rich in β-glucans and heteropolysaccharides with various bioactive properties, including antioxidant activity (Ayimbila & Keawsompong, 2021). The fruiting bodies of these mushrooms are cultivated as sources of polysaccharides. The extracted polysaccharides were characterized and subsequently used in batch fecal fermentation experiments to assess their effects on the human gut bacterial community. Our findings support the potential application of polysaccharides derived from edible mushrooms of the *Lentinus* species in modulating human gut bacterial communities.

## Materials & Methods

### Ethical approval

The procedure for the collection and use of fecal samples from volunteers was approved by the Ubon Ratchathani University Ethics Committee for Human Research with permission number UBU-REC-35/2566. Written informed consent was obtained from all participants prior to sample collection in accordance with the study protocol.

### Collection of wild edible mushroom mycelia

Mycelia were collected from two wild mushroom isolates, PB1 and LP1. Mushroom mycelium PB1 was originally isolated from the Piboonmungsahan District, Ubon Ratchathani, Thailand. LP1 refers to another isolate obtained from a forest at Ubon Ratchathani University, located in the Warin Chamrab District of Ubon Ratchathani, Thailand. The mycelia were submerged in sterile distilled water and maintained at ambient temperature.

### Fungal genomic DNA extraction

The stock mycelia of each mushroom were regrown on potato dextrose agar (PDA) (Oxoid, Basingstoke, UK) at 32 °C for 7 d. Mushroom mycelia were harvested and used for genomic DNA extraction using the Prep Fungi/Yeast Genomic DNA Extraction Mini Kit following the manufacturer’s instructions (Favorgen Biotech Corp., Ping Tung, Taiwan). The quality of the extracted DNA was verified by measuring absorbance at 260 and 280 nm. A ratio of A260/280 between 1.8 and 2.0 was accepted for further use. The extracted DNA was preserved at −20 °C until use.

### Species identification of mushrooms

Wild mushroom species were identified using polymerase chain reaction (PCR) to amplify the 28S rRNA gene, followed by nucleotide sequencing and analysis. Oligonucleotide primers and amplification conditions were as described previously (Raja et al., 2017). The chemicals for PCR were obtained from the AllTaq™ PCR Core Kit (Qiagen, Hilden, Germany). Amplified products (amplicons) were verified using gel electrophoresis. Amplicons were purified using a HiYield™ Gel/PCR DNA Fragment Extraction Kit (RBC Bioscience, Taiwan). Nucleotide sequencing was conducted at the ATGC (Thailand Science Park, Thailand) using the chain-termination DNA technique. The sequencing results were compared with fungal 28S rDNA sequences from the National Center for Biotechnology Information (NCBI) database using extremely comparable sequence (megablast) parameter settings. Molecular Evolutionary Genetics Analysis Version 11 (MEGA11) uses the neighbor-joining method with bootstrap values for 1,000 replicates to construct a phylogenetic tree (Tamura, Stecher & Kumar, 2021).

### In-house cultivation of mushrooms

Purified mycelia of both PB1 and LP1, previously identified at the species level, were grown on PDA (Oxoid) at 32 °C for 7 d. After incubation, mycelia measuring 1  ×  1 centimeters (cm) were cut from the PDA and transferred to a ripe millet-containing bottle. The culture was incubated at 32 °C for 14 d. A total of 8–10 millet seeds enveloped in mushroom mycelia were inserted into four designated locations on the sides of sawdust plastic bags, which were then incubated at ambient temperature in a mushroom cultivation house. Water was sprayed onto the bags twice daily to maintain a relative humidity of approximately 70–90%. Fruiting bodies were harvested 14–28 d after inoculation. All mushrooms were visually inspected for morphological uniformity, including cap morphology and coloration, prior to processing. Harvested samples were pooled and homogenized prior to polysaccharide extraction. Mushroom cultivation was conducted at Pundee Mushroom Farm, Warinchamrap District, Ubon Ratchathani Province, Thailand.

### Extraction of crude polysaccharides

Crude polysaccharides were extracted from each mushroom as described in our previous report, with slight modifications (Panya et al., 2024). The modified procedure included an initial extraction step, in which a mixture of mushroom powder and water was incubated at 90 °C in a sonication bath for 30 min. The mixture was incubated in a water bath without sonication at the same temperature for 3 h. After cooling at 20–25 °C), the mixture was centrifuged at 9,000 × *g* for 20 min. The clear supernatant was collected and mixed with a butanol–chloroform solution at a 1:1 ratio (clear supernatant:butanol–chloroform = 1:1) to remove residual proteins and improve the purity of the crude polysaccharide extract. The subsequent steps followed the procedure described by Panya et al. (2024). The crude polysaccharides extracted from *L. squarrosulus* PB1 and *L. polychrous* LP1 are hereafter referred to as LS and LP, respectively. The extraction yields were calculated as previously described (Panya et al., 2024).

### Chemical composition of the crude polysaccharides

The chemical composition of the crude polysaccharides was analyzed to determine the total carbohydrate, reducing sugar, protein, and phenolic content, as described in previous reports. The total carbohydrate content of the crude polysaccharides was measured using the phenol–sulfuric acid method (Masuko et al., 2005), with absorbance recorded at 490 nm using D-(+)-glucose as the standard solution. Reducing sugars were quantified using the 3,5-dinitrosalicylic acid method (Miller, 1959) at 540 nm, using glucose as the standard solution. The protein content of the extracted crude polysaccharides was assessed as previously described (Thetsrimuang et al., 2011). The absorbance of the reaction mixture was measured at 595 nm using a microplate reader. Bovine serum albumin (Sigma-Aldrich, St. Louis, MO, USA) was used to prepare a standard protein solution (Thetsrimuang et al., 2011). Total phenolic compounds were determined using the Folin–Ciocalteu method (Aliaño González et al., 2022), with absorbance measured at 765 nm, and gallic acid was used to prepare the standard solution. All experiments were performed in triplicate.

### Simulation of human gastrointestinal digestion

**Figure 1:**
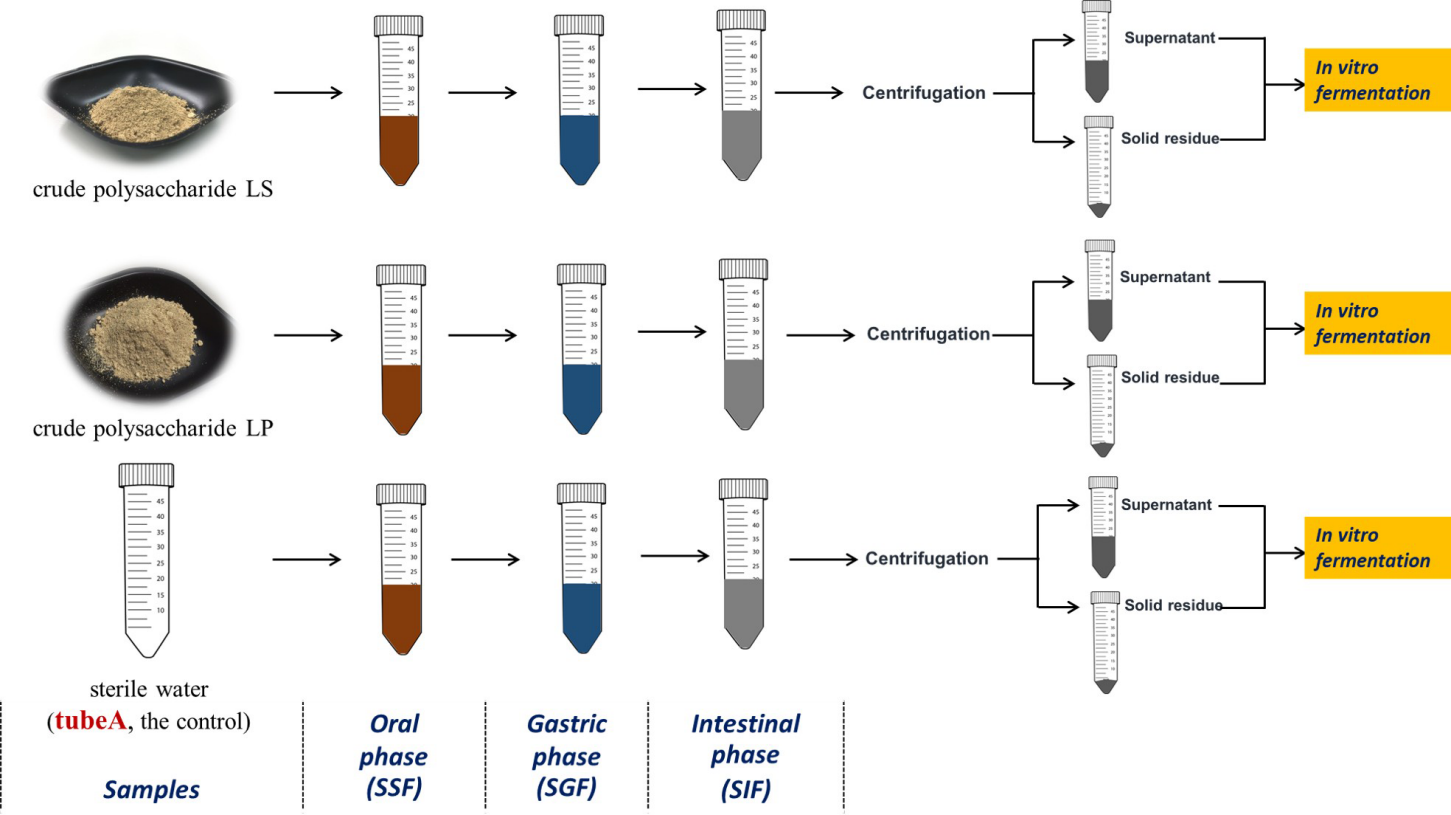
The simulated human gastrointestinal digestion process prior to *in vitro* fermentation. Schematic representation of the simulated human gastrointestinal digestion process prior to *in vitro* fermentation. The digestion simulation consisted of three consecutive phases: the oral phase (SSF; simulated salivary fluid), gastric phase (SGF; simulated gastric fluid), and intestinal phase (SIF; simulated intestinal fluid). During each phase, the respective simulated fluid was added sequentially to the crude polysaccharide samples from *L. squarrosulus* (LS) and *L. polychrous* (LP). After completion of the intestinal phase, the mixtures were centrifuged to separate the supernatant and solid residue, both of which were subsequently used for the *in vitro* fecal fermentation. A digestion control (tubeA) was prepared in parallel using sterile water instead of crude polysaccharides. The figure was created by the authors using Adobe Illustrator CS6.

The simulation of the human gastrointestinal ingestion conditions followed that of a previous study (Minekus et al., 2014). The digestive process comprises three sequential phases: oral, gastric, and intestinal, which are briefly illustrated in Fig. 1. Table 1 lists the chemical solutions and buffers used for the digestion process. For the oral phase, 5 g of crude polysaccharide powder, either LS or LP, was mixed with simulated salivary fluid supplemented with 1,500 U/mL α-amylase (α-amylase from human saliva type XIII-A, lyophilized powder, 300–1,500 units/mg protein, product no. A1031-1KU; Sigma Aldrich, St. Louis, MO, USA). The solution was vortexed and incubated at 37 °C for 2 min. For the gastric phase, the solution was further mixed with simulated gastric fluid supplemented with 25,000 U/mL pepsin (pepsin from porcine gastric mucosa ≥3,200 units/mg protein, Sigma-Aldrich product no. P6887). The solution was vortexed and incubated at 37 °C for 2 h. The solution was vortexed for 15 min during incubation. After incubation, simulated intestinal fluid containing 800 U/mL porcine pancreatin (Cat. No. P7545; Sigma Aldrich, St. Louis, MO, USA) and bile salts (product no. B8756; Sigma Aldrich, St. Louis, MO, USA) was added to the solution. The solution was vortexed and incubated at 37 °C for 2 h. In the final step, the solution tubes were centrifuged at 9,000 × *g* for 10 min to separate the supernatant from the solid residue, which was then used for fermentation. To prepare the control (tube A), sterile water was used instead of crude polysaccharides during digestion. The percentage of hydrolysis (H%) was calculated as the ratio of reducing sugar released to the total sugar content using the following equation, following the method described by Ayimbila et al. (2022):

\begin{eqnarray*}\text{Percentage hydrolysis}~ \left( \% \right) = \frac{\text{Reducing sugar relaeased}~(\mathrm{final}-\text{initial sugar})}{\text{Total sugar content}-\text{initial reducing sugar}} \times 100. \end{eqnarray*}

### Selection and allocation of volunteers for feces donation

People living in the Khoo Mueang Subdistrict, Warinchamrap District, Ubon Ratchathani Province, were invited to volunteer. The rationale and aim of this study, as well as the criteria for selecting volunteers, were thoroughly explained to potential volunteers. The criteria for selecting volunteers were as follows: (1) age 18–35 years; (2) no recognized gastrointestinal diseases; and (3) no antibiotic, probiotic, or prebiotic consumption for at least six months before donating the fecal sample.

**Table 1:** Chemical components in simulated digestive solutions.

Chemicals	Simulated digestive solutions and their final concentrations (mmol/l)		
	Simulated salivary fluid (SSF)	Simulated gastric fluid (SGF)	Simulated intestinal fluid (SIF)
KCl	15.10	6.90	6.80
KH_2_PO_4_	3.70	0.90	0.80
NaHCO_3_	13.60	25.00	85.00
NaCl	–	47.20	38.40
MgCl_2_(H_2_O)_6_	0.15	0.10	0.33
(NH_4_)_2_CO_3_	0.06	0.50	–
CaCl_2_(H_2_O)	1.50	0.15	0.60
Final pH	7.00	3.00	7.00

### Fecal slurry preparation

Fecal samples were obtained from four registered volunteers without any sex restrictions. Fecal samples were collected in sterile bottles and kept under microaerobic conditions in an anaerobic jar (Schuett Biotec, Göttingen, Germany) containing an oxygen absorber–CO_2_ generator system (Campygen; Oxoid) and immediately transported to the Microbiology Laboratory, College of Medicine and Public Health, Ubon Ratchathani University, Warinchamrap, Ubon Ratchathani, Thailand. After arriving at the laboratory, the fecal samples were placed into the BACTRON anaerobic chamber (85% N_2_, 10% CO_2_, 5% H_2_) (Sheldon Manufacturing Inc., Cornelius, OR, USA). All fecal samples were collected from individual volunteers on the morning of the same day. The fecal slurry was prepared as previously described (Pérez-Burillo et al., 2021). Briefly, 10 g of each fecal sample was weighed and thoroughly mixed in a 50-mL tube by shaking continuously at 2,000 rpm for 5 min. Thirty-two grams of mixed feces was aliquoted and dissolved in phosphate buffer (pH 7.2; Sigma-Aldrich, St. Louis, MO, USA) to obtain a final concentration of 32% (w/v) feces, which was immediately used for the fermentation process. The entire preparation was performed under anaerobic conditions in an anaerobic chamber as described above, and the resulting fecal slurry was immediately used in the fermentation process.

### *In vitro* batch fermentation

**Figure 2:**
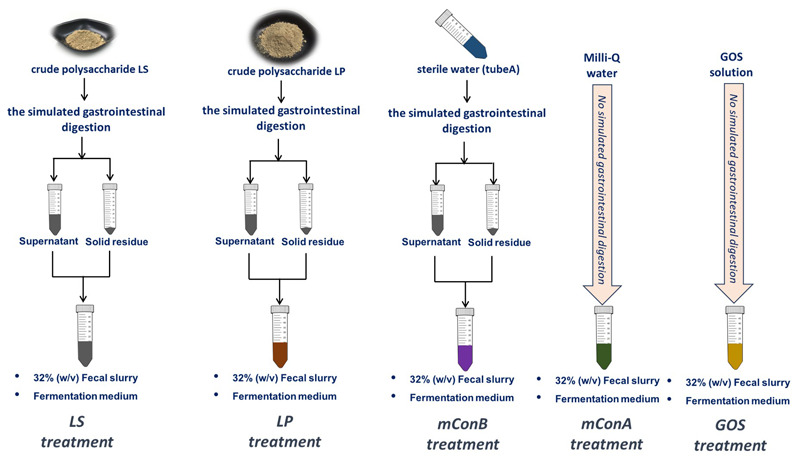
The *in vitro* batch fermentation setup using digested crude polysaccharides from *Lentinus* species. Schematic representation of the *in vitro* batch fermentation setup using digested crude polysaccharides from *Lentinus* species. Crude polysaccharides from *L. squarrosulus* (LS) and *L. polychrous* (LP) that had undergone simulated gastrointestinal digestion were used as substrates for fecal fermentation. The experiment consisted of five treatments, including LS, LP, mConB, mConA, and GOS. Two control treatments were included: mConA, which used Milli-Q water instead of the test substances, and mConB, which used the solid residue and supernatant from tubeA (the digestion control). Galactooligosaccharide (GOS) was used as a reference prebiotic. All fermentation mixtures were prepared in 50 mL sterile tubes containing 32% (w/v) fecal slurry and fermentation medium, vortexed thoroughly, and incubated under anaerobic conditions in a BACTRON anaerobic chamber. The figure was created by the authors using Adobe Illustrator CS6.

The crude polysaccharides, LS and LP, that had undergone simulated gastrointestinal digestion were fermented with the fecal slurry of volunteers using the batch fermentation method (Fig. 2). Two control sets of mConA (Milli-Q water was used instead of a substrate to minimize background nutrient interference) were prepared as a fermentation medium with Milli-Q water. mConB (a solid residue and a supernatant from tube A from the simulation of the human gastrointestinal digestion step) was included in the experiment. As previously described by Pérez-Burillo et al. (2021), approximately 10% of the absorbable content is not absorbed in the small intestine *in vivo* and reaches the large intestine. Therefore, adding 10% (v/v) of the soluble fraction (supernatant) derived from the simulated gastrointestinal digestion step to the solid residue further mimics colonic conditions. Undigested galactooligosaccharides (GOS) were used as reference prebiotics for fermentation. Moreover, the original fecal slurry (BL) from volunteers that was not subjected to *in vitro* fermentation was included to represent the baseline bacterial community at 0 h after fermentation. The total number of samples used in this study is summarized in Fig. S1. Each sample was analyzed in quadruplicate, except for the GOS control group, because two sample tubes were lost during fermentation; thus, only two GOS samples were retained. This was the same for the original fecal slurry solution (BL). Three samples of BL were used. In total, 21 samples were analyzed in this study: three for BL, four for mConA, four for mConB, two for GOS, four for LP, and four for LS. Table 2 shows the composition of each testicular sample, which is also referred to as treatment. All solutions were prepared in 50-mL sterile tubes and mixed by vortexing. The tube caps were slightly opened and placed in a BACTRON anaerobic chamber at 37 °C until the fermentation was completed. Pre- (0 h) and post-fermentation (24 h) samples were collected for genomic DNA extraction and nucleotide sequencing, targeting the hypervariable V3–V4 regions of the bacterial 16S rRNA gene.

**Table 2:** Components of each treatment for *in vitro* batch fermentation.

Treatments	Components			
	**Fermentation** ** medium** ^a^ ** (ml)**	**32% (w/v) Fecal slurry** ** (ml)**	**Supernatant from the simulation of human gastrointestinal digestion** ** (µl)**	**Solid residue from the simulation of human gastrointestinal digestion** ** (g)**
Crude polysaccharide LS	7.5	2	350	0.5
Crude polysaccharide LP	7.5	2	350	0.5
GOS	7.5	2	0.5 g GOS was dissolved in 1.0 ml sterile water and filtered through a 0.2 µm syringe filter^b^	
mConA	7.5	2	0.85 ml Milli-Q water	
mConB	7.5	2	350 µl from tubeA^c^	0.5 g from tubeA^c^

**Notes.**

a The fermentation medium composed of 2.0 g yeast extract, 0.02 g K_2_HPO_4_, 0.005 g CaCl_2_, 1.0 g NaHCO_3_, 0.05 g NaCl, 1.0 g peptone, 0.005 g MgSO_4_, 0.23 g cysteine-HCl, 0.5 g resazurin, 0.01 g hemin, 1.0 mL Tween 80, 0.25 g bile salt, and 5 µL vitamin K1, which is a sugar-free medium, was prepared according to the method of Pérez-Burillo et al. (2021). After sterilization, the fermentation medium was bubbled with CO_2_ for 1 min to eliminate dissolved O_2_ prior to use.

b GOS was not subjected to the simulation of human gastrointestinal digestion.

c tubeA is obtained from the step of the simulation of human gastrointestinal digestion.

### Extraction and qualification of genomic DNA

Total genomic DNA was extracted from each sample using the ZymoBIOMICS DNA Miniprep Kit (Zymo Research Corp., Irvine, CA, USA), following the manufacturer’s protocol. The quality and quantity of the genomic DNA were measured using a Qubit Fluorometer (Thermo Fisher Scientific, Waltham, MA, USA). Conventional PCR with the primer pair 27F (5′-AGAGTTTGATCCTGGCTCAC-3′) and 1492R (5′-GGTTACCTTGTTACGCTT-3′) was used to amplify the target 16S rRNA gene of bacteria. Amplification products were verified using agarose gel electrophoresis. The extracted DNA was stored at −20 °C until use.

### 16S rRNA gene amplicon sequencing and bioinformatics analysis

The V3–V4 region of the bacterial 16S rRNA gene was amplified using primers 341F and 806R (341F, 5′-CCTAYGGGRBGCASCAG-3′, 806R, 5′-GGACTACNNGGGTATCTAAT-3′) with Phusion High-Fidelity polymerase, purified with AMPure XP beads, and sequenced using Illumina adapters. Libraries were pooled equimolarly and sequenced on an Illumina NovaSeq 6000 platform to generate 250 bp paired-end reads. Raw sequences were processed in QIIME 2 (Bolyen et al., 2019), where reads were denoised, merged, and chimera-filtered using DADA2 (Callahan et al., 2016) to obtain high-resolution amplicon sequence variants (ASVs). Taxonomic classification was performed using a Naïve Bayes classifier trained using the SILVA v138 reference database. To standardize the data across samples, the ASV table was rarefied to a uniform sequencing depth to reduce biases from unequal library sizes, and taxonomic counts were converted to relative abundances to allow for a direct comparison of bacterial community composition (Callahan, McMurdie & Holmes, 2017). For alpha diversity (observed features, Chao1, Shannon, Simpson) and beta diversity, the feature table was rarefied to a uniform sequencing depth (selected from rarefaction curves); beta diversity distance matrices were computed using Bray–Curtis and weighted/unweighted UniFrac, followed by principal coordinate analysis for ordination and the unweighted pair-group method with arithmetic mean clustering (on weighted UniFrac) (Backeljau et al., 1996; Li et al., 2013). Principal component analysis is a Euclidean-based dimensionality-reduction method that captures the major variance in multidimensional data, allowing samples with similar community compositions to cluster closely on a two-dimensional plot (Jolliffe, 1986).

### Statistical analysis

Alpha diversity differences were assessed using the Kruskal–Wallis test, with statistical significance set at *p* < 0.05 (Bolyen et al., 2019). Beta diversity differences were evaluated using PERMANOVA based on weighted UniFrac distance matrices. Pairwise comparisons of relative taxonomic abundance at the phylum, family, and genus levels were performed using independent *t*-tests (*p* < 0.05). Comparisons of the Firmicutes/Bacteroidota and Blautia/Bacteroides relative abundance ratios between 0 and 24 h under the LS and LP treatments were performed using the Wilcoxon signed-rank test. Similarly, differences in pH values between 0 and 24 h after fermentation with mConA, mConB, GOS, LS, and LP treatments were evaluated using the Wilcoxon signed-rank test. Statistical significance was set at *p* < 0.05.

## Results

### Species identification of mushrooms

**Figure 3:**
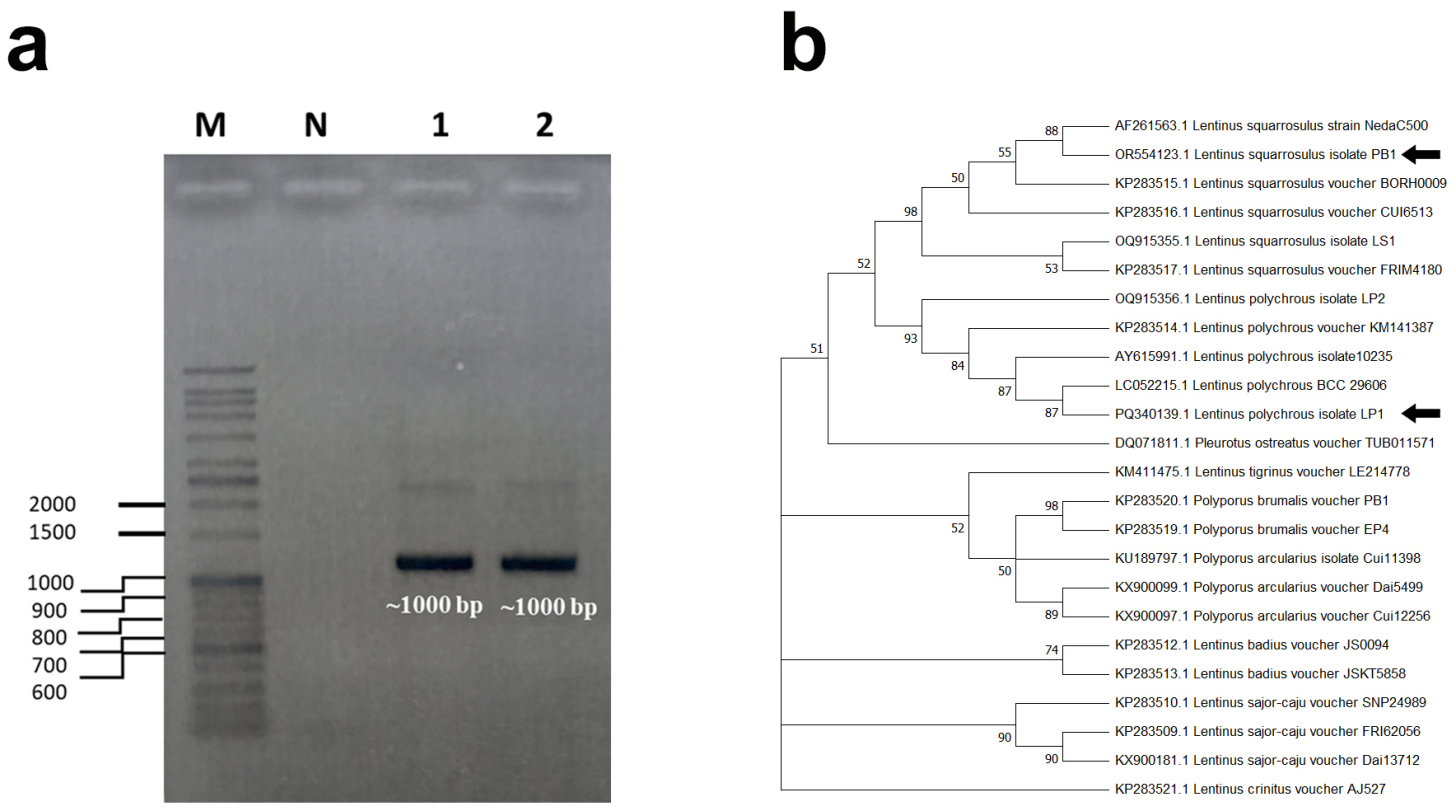
Molecular identification of mushrooms. (A) Ethidium bromide-stained agarose gel exhibiting LSU-28S rRNA amplified from *L. squarrosulus* PB1 and *L. polychrous* LP1. Lane M had VC DNA ladder. Lanes 1 and 2 contained PB1 and LP1 LSU-28S rRNA gene-amplified products. Lane N contains PCR product without DNA template. DNA ladder’s base pairs (bp) are shown on gel. (B) Taxa evolutionary relationships are identified using LSU-28S rRNA gene data and neighbor-joining method. Each node has bootstrap support above 50%. Evolutionary analyses were conducted using MEGA11. *Pleurotus ostreatus* was used as an outgroup. The arrow behind taxonomy names indicates LSU-28S rRNA of *L. squarrosulus* PB1 and *L. polychrous* LP1.

The PCR results indicated that the amplified product was approximately 1,000 bp and was obtained from the mushrooms with the codes PB1 and LP1 (Fig. 3A). Nucleotide sequencing analysis of the amplified products from mushrooms PB1 and LP1 revealed nucleotide sequences of 1,075 and 1,045 bp, respectively. The 1,045 bp nucleotide sequence of LP1 showed 100% identity with the nucleotide sequence of the large subunit of the 28S ribosomal RNA gene (LSU-28S rRNA) of the mushroom *L. polychrous* voucher KM141387 (GenBank accession no. KP283514.1). The 1,075 bp nucleotide sequence of PB1 is identical to the LSU-28S rRNA of the mushroom *L. squarrosulus* voucher FRIM4180 (GenBank accession no. KP283517.1). Phylogenetic analysis (Fig. 3B) showed that the LSU-28S rRNA sequences of PB1 and LP1 clustered closely with those of *L. squarrosulus* and *L. polychrous*, respectively. Based on these results, the PB1 and LP1 strains were identified as *L. squarrosulus* and *L. polychrous*, respectively, and designated as *L. squarrosulus* PB1 and *L. polychrous* LP1.

### Cultivation of mushrooms

**Figure 4:**
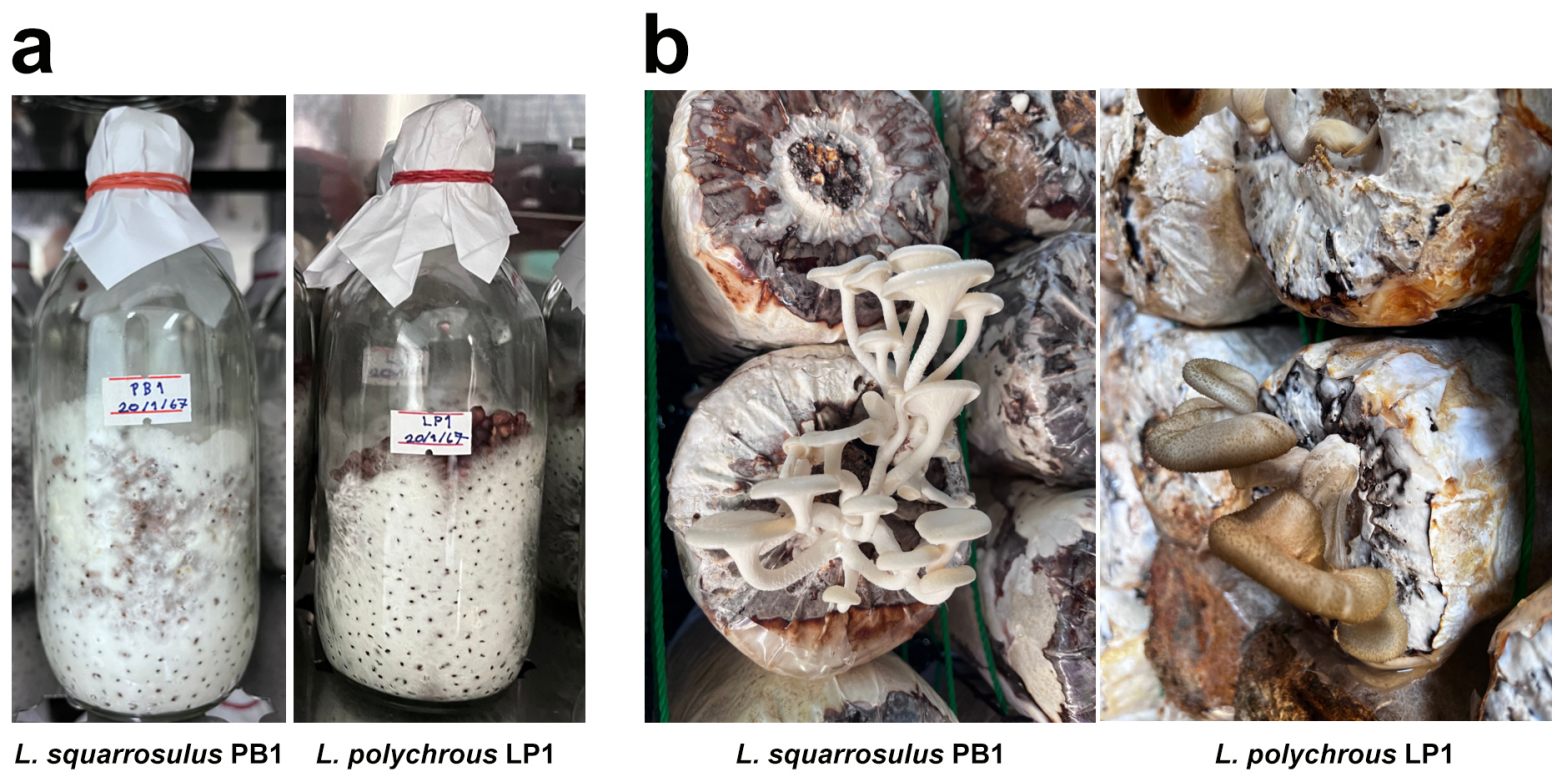
Cultivation of mushrooms *L. polychrous* LP1 and *L. squarrosulus* PB1. (A) Mycelia grown on millet grains for 14 days. (B) Fruiting bodies of mushrooms in sawdust plastic bags after 14 days of cultivation.

Cultivation of mushroom mycelia in millet seeds showed that after 14 d of incubation, *L. polychrous* LP1 and *L. squarrosulus* PB1 developed white mycelia in the bottles (Fig. 4A). Mushrooms grown in sawdust plastic bags showed that *L. polychrous* LP1 had brownish funnel-shaped gills, whereas *L. squarrosulus* PB1 had white, button-like caps (Fig. 4B). The fruiting bodies of the mushrooms were harvested approximately 14 d after cultivation (when the caps started to flatten). Two weeks after mushroom harvesting, each type of mushroom produced a yield of approximately 200–300 g/one sawdust plastic bag.

### Components in extracted crude polysaccharides

**Figure 5:**
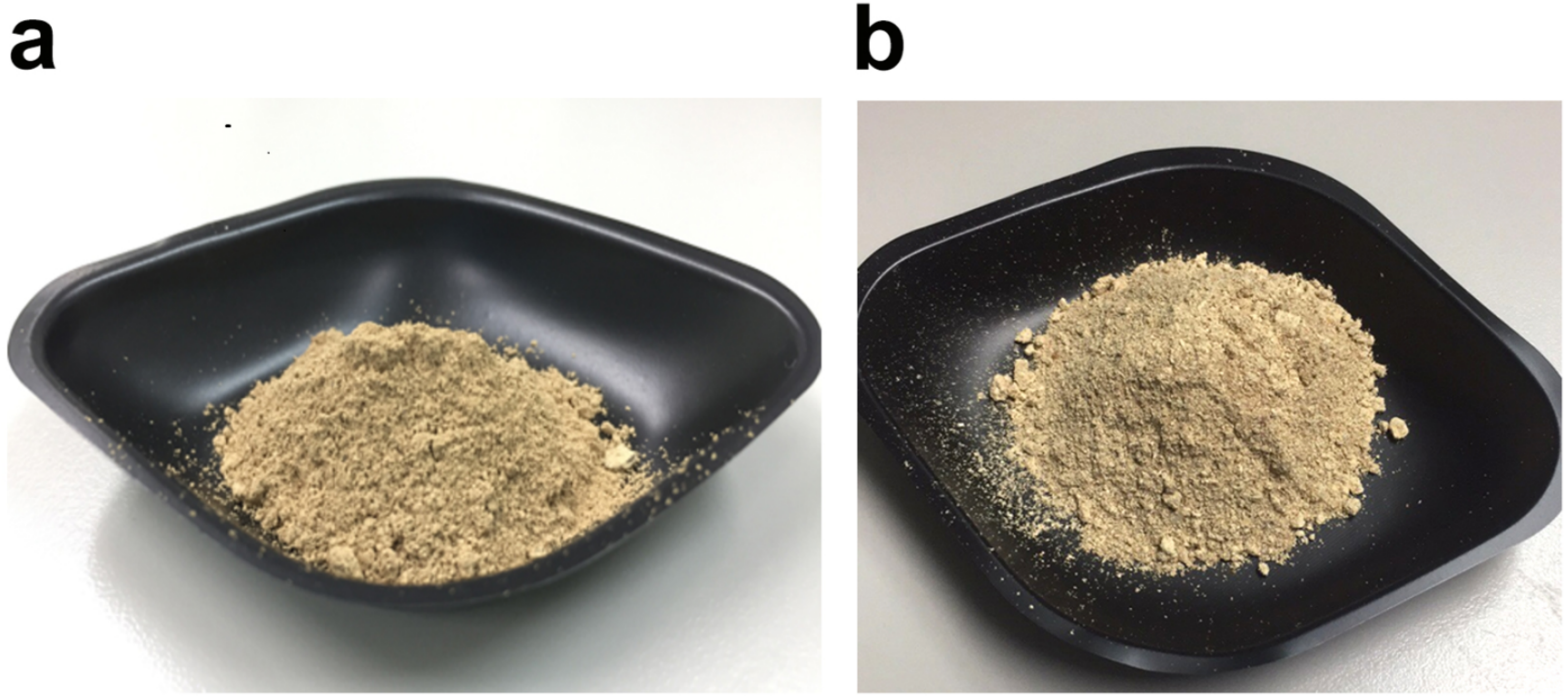
Appearance of crude polysaccharide LS from *L. squarrosulus* PB1 (A) and LP from *L. polychrous* LP1 (B).

After extraction, the crude polysaccharides derived from *L. squarrosulus* PB1 (designated LS) and *L. polychrous* LP1 (designated LP) were light brown (Fig. 5). As shown in Table 3 and Table S1, the major components of both LS and LP were carbohydrates with low amounts of proteins and reducing sugars. The extraction yields of crude polysaccharides (LS and LP) were 7.90 ± 0.04 and 9.28 ± 0.03, respectively. The polysaccharide contents of LS and LP were approximately 84.65% and 85.79% of total carbohydrates, respectively. Moreover, both the LS and LP contained small quantities of phenolic compounds.

**Table 3:** Yield of crude polysaccharides and amount of total carbohydrates, reducing sugars, polysaccharides, total proteins, and phenolic compounds present in crude polysaccharides LS and LP (*n* = 3).

Crude polysaccharides	Yield (%)	Total carbohydrates (mg/g)	Reducing sugars (mg/g)	Polysaccharides (mg/g)	Total proteins (mg/g)	Phenolic compounds (mg/g)
LS	7.90 ± 0.04	546.09 ± 13.04	83.82 ± 4.32	462.27 ± 13.05	19.08 ± 3.15	34.38 ± 4.53
LP	9.28 ± 0.03	612.75 ± 26.21	87.09 ± 3.47	525.66 ± 26.21	28.46 ± 1.32	67.72 ± 2.88

**Notes.**

The value is expressed as the mean ± S.D. of triplicate experiments.

### Resistance of crude polysaccharides to simulated human gastrointestinal digestion

**Figure 6:**
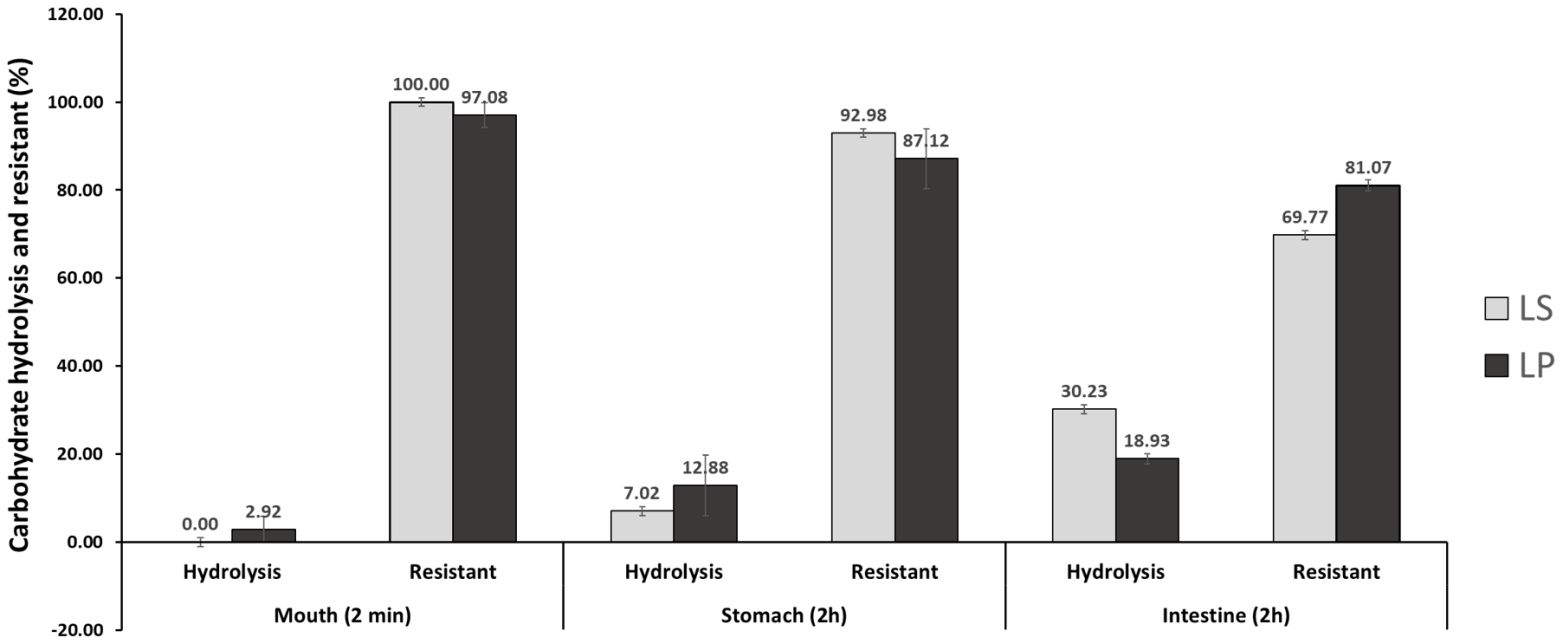
Carbohydrate hydrolysis and resistance of crude polysaccharides from *L. squarrosulus* (LS) and *L. polychrous* (LP) during simulated digestion. The percentage of carbohydrate hydrolysis and resistant fractions were determined after sequential exposure to simulated mouth (2 min), stomach (2 h), and intestine (2 h) digestion phases. Data represent the mean ± SD of one independent experiment performed in triplicate.

As shown in Fig. 6 and Table S2, the crude polysaccharides from *L. polychrous* (LP) and *L. squarrosulus* (LS) exhibited high resistance to digestion, maintaining 81.07% and 69.77% of their polysaccharide structures, respectively, after 4 h of sequential exposure to simulated oral, gastric, and intestinal conditions.

### Characteristics of volunteers for feces donation

Stool samples were collected from four volunteers. All volunteers were male, with an average age of 21.5 years. Their general interview revealed that they consumed a balanced diet, had no recognized chronic diseases, were not undergoing treatment for gastrointestinal diseases, had no history of infectious diseases, and had no history of antibiotic use in the past six months. All the volunteers were willing to participate in this study.

### Verification of genomic DNA extracted before and after fermentation

Using conventional PCR followed by agarose gel electrophoresis, specific DNA fragments of approximately 1,500 base pairs were identified (indicated by the arrow in Fig. S2A) encompassing the 16S rRNA gene, were amplified in all fermentation treatments. Moreover, the quantity and purity of genomic DNA, verified using a NanoDrop spectrophotometer (Thermo Fisher Scientific Inc., Wilmington, DE, USA), showed acceptable amounts and quality that met the acceptance criteria for 16S metagenomic DNA sequencing (Table S3).

### Quality of nucleotide sequencing output

Sequencing data output showed that the average number of paired-end raw reads per sample was 203,341 (Table S4). After quality filtering and chimeric sequence removal, 161,046 effective reads (Nochime) were retained, with an average read length of 417 nucleotides. Additionally, sequencing quality was consistently high across all samples, with Q20 scores exceeding 97.91% and Q30 scores exceeding 93.60%, indicating excellent base-calling accuracy and strong suitability for downstream microbiome analyses.

### Detection and analysis of bacterial species present in samples

**Figure 7:**
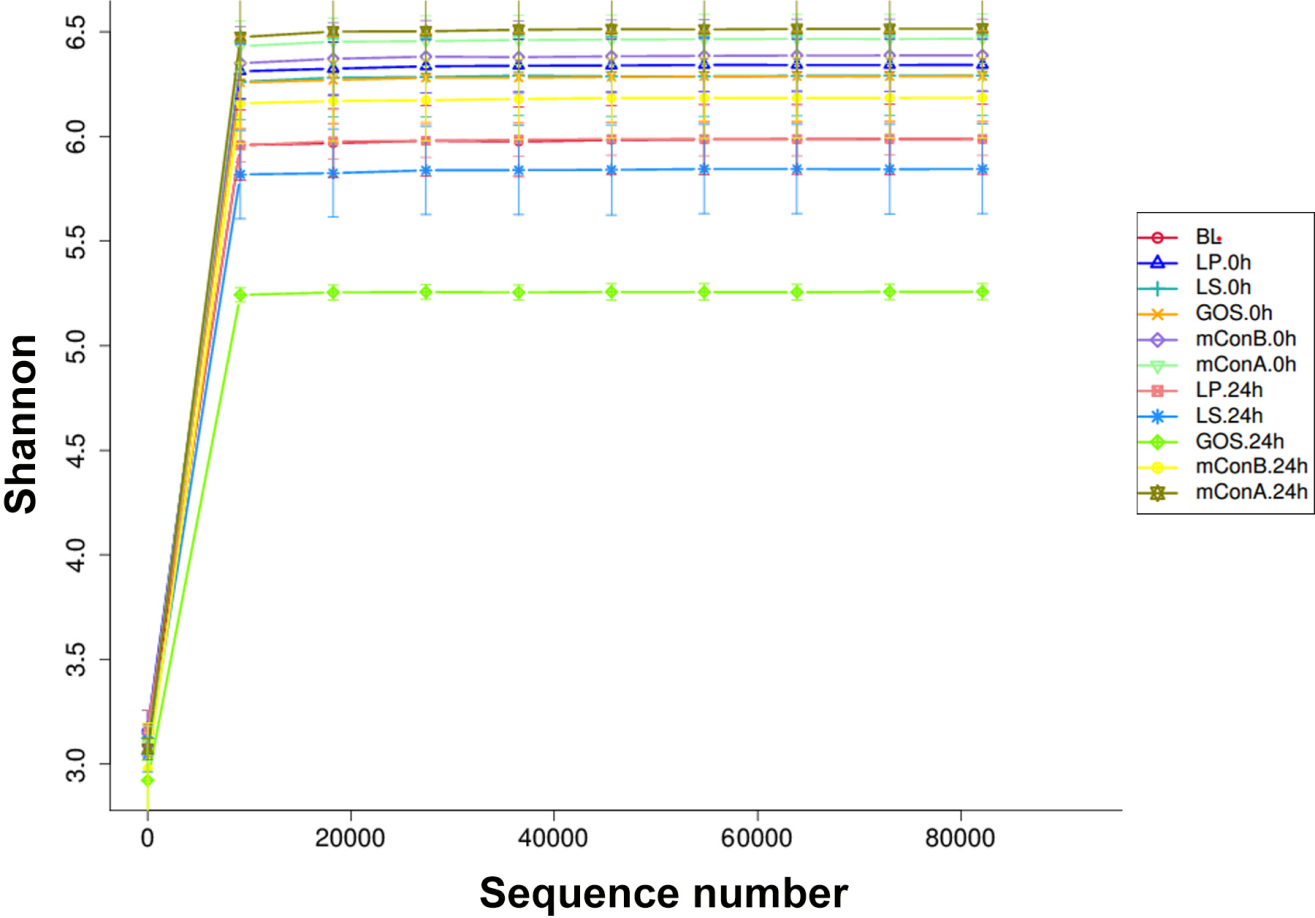
Shannon refraction curve analysis. Sequence number is on *X*-axis, and observed amplicon sequence variants (ASVs) are on *Y*-axis.

Shannon rarefaction curve analysis (Fig. 7) showed that although the number of sequence reads increased progressively with deeper sampling, the Shannon diversity values (Table S5) for all treatments quickly reached a plateau. This flattening pattern indicated that additional sequencing would not reveal further bacterial diversity and that nearly all ASVs present in the samples were captured. The consistent plateau across all groups confirmed that the sequencing depth was sufficient and that the diversity estimates obtained in this study were reliable and suitable for further microbiome analyses.

### Crude polysaccharides altered the diversity of gut microbiota

**Figure 8:**
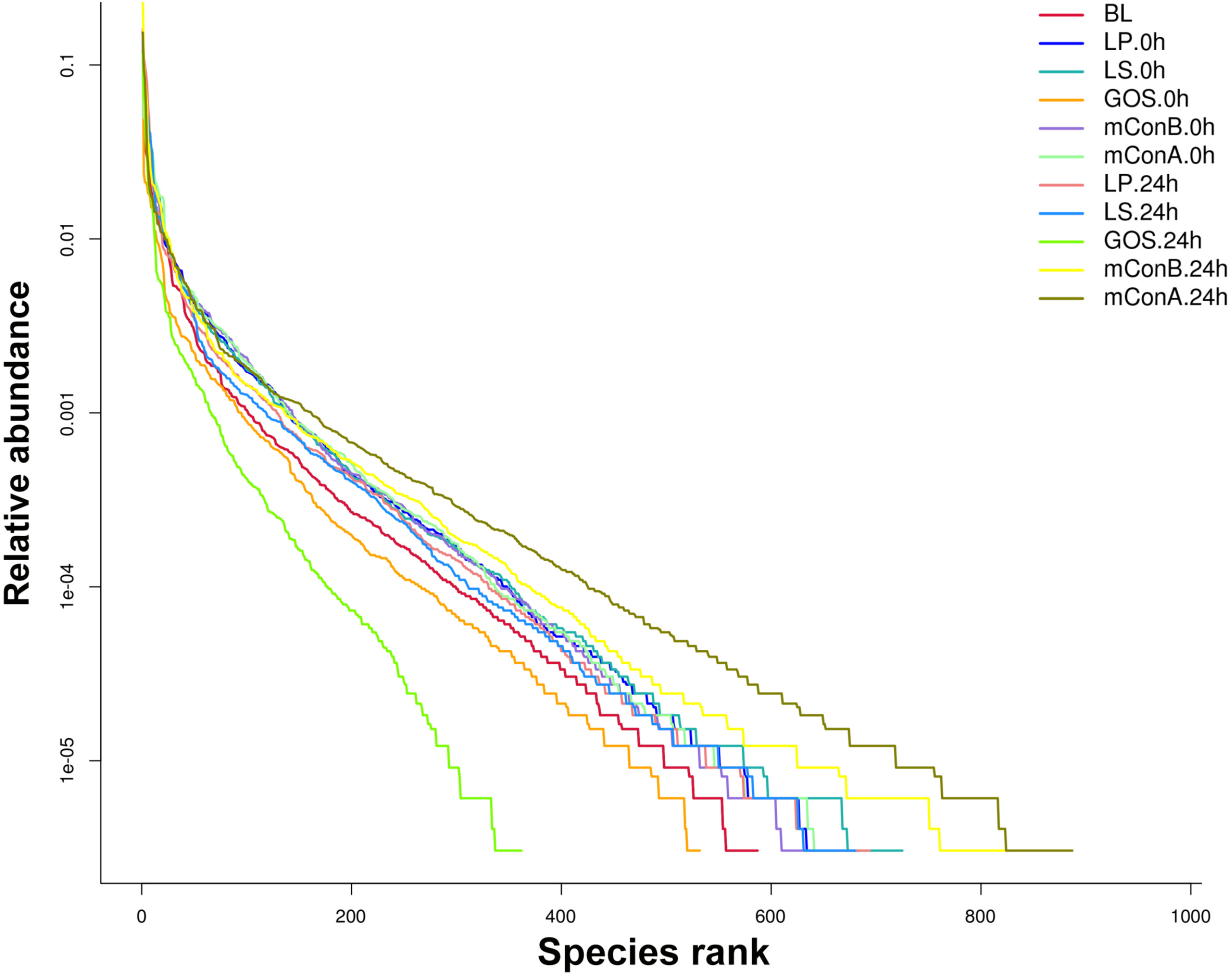
Species rank abundance curve. Relative abundance of each species is plotted against its rank order.

**Figure 9:**
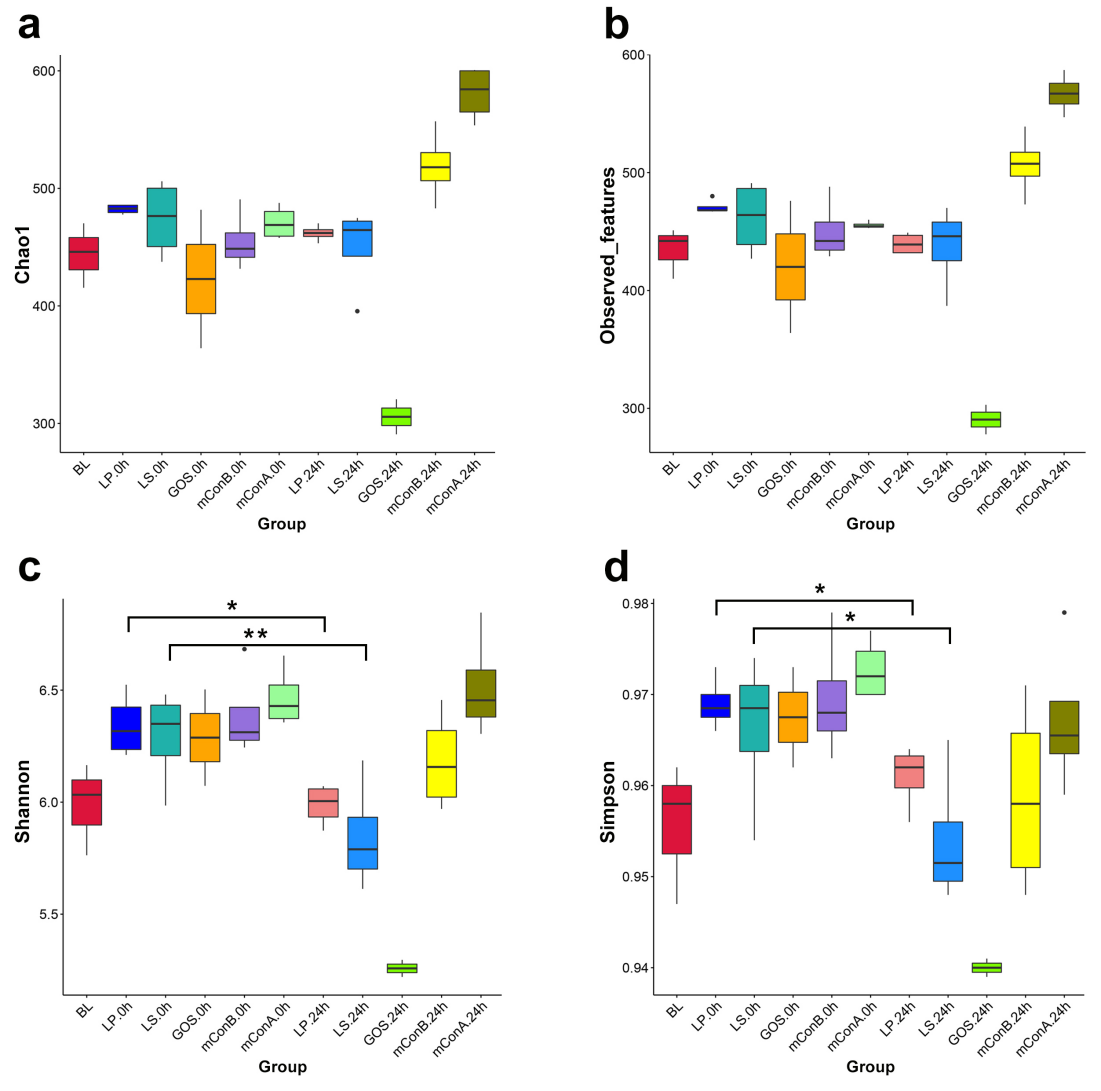
Alpha diversity indices of bacterial composition of treatments. (A) Chao1. (B) Observed feature. (C) Shannon. (D) Simpson. Horizontal axis of boxplot represents groups, while vertical axis represents corresponding alpha diversity index value. Kruskal–Wallis test was performed for statistical analysis. Significant differences of *p* < 0.05 and *p* < 0.01 are indicated by ∗ and ∗∗, respectively.

The rank abundance curve analysis (Fig. 8) demonstrated the diversity (species richness and evenness) of the gut microbiota within the treatment groups. Species richness represents the total number of species, whereas species evenness represents the distribution of individuals among the species. After 24 h of fermentation, mConA.24 h exhibited the highest species richness and evenness, as evidenced by its gradual slope and extended horizontal reach on the rank abundance curve. Conversely, while mConB.24 h maintained a relatively diverse profile, it displayed a steeper slope compared to mConA.24 h. The reference prebiotic GOS (GOS.24h) treatment had the lowest species rank compared with the other treatments, indicating the dominance of the least abundant bacterial species in the sample. Similarly, the variety of bacterial species was lower in the groups treated with LP (LP.24h) and LS (LS.24h) after 24 h of fermentation than in the samples collected before fermentation (0 h). Additionally, the alpha indices (Chao1 and observed features, which count all species, including rare ones, and Shannon and Simpson indices, which measure community diversity) are displayed in Fig. 9. The number of bacterial species was slightly reduced after fermentation with the crude LP and LS (Figs. 9A and 9B), but this decrease was not statistically significant (*p* > 0.05) (Table S6). The evenness of the bacterial species was significantly reduced after fermentation (Figs. 9C and 9D), indicating that LP and LS supported the growth of specific bacterial species.

### Beta diversity analysis shows a clear distinction between treatment groups

**Figure 10:**
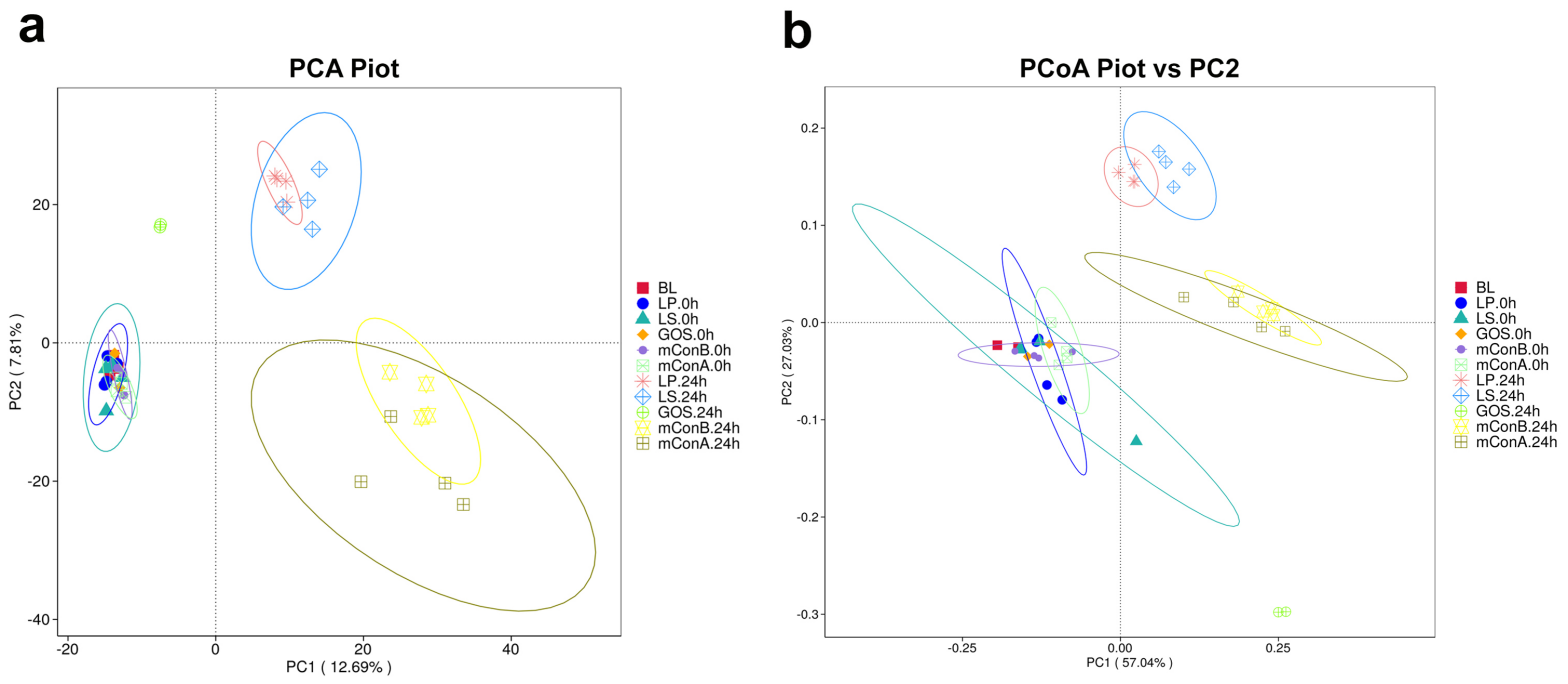
Beta diversity analysis of bacterial composition of treatments. (A) Principal component analysis (PCA). (B) Principal coordinate analysis (PCoA). PCoA is based on weighted UniFrac distance. Each point represents sample, plotted with principal component on *X*-axis and another principal component on *Y*-axis, which was colored by group.

**Figure 11:**
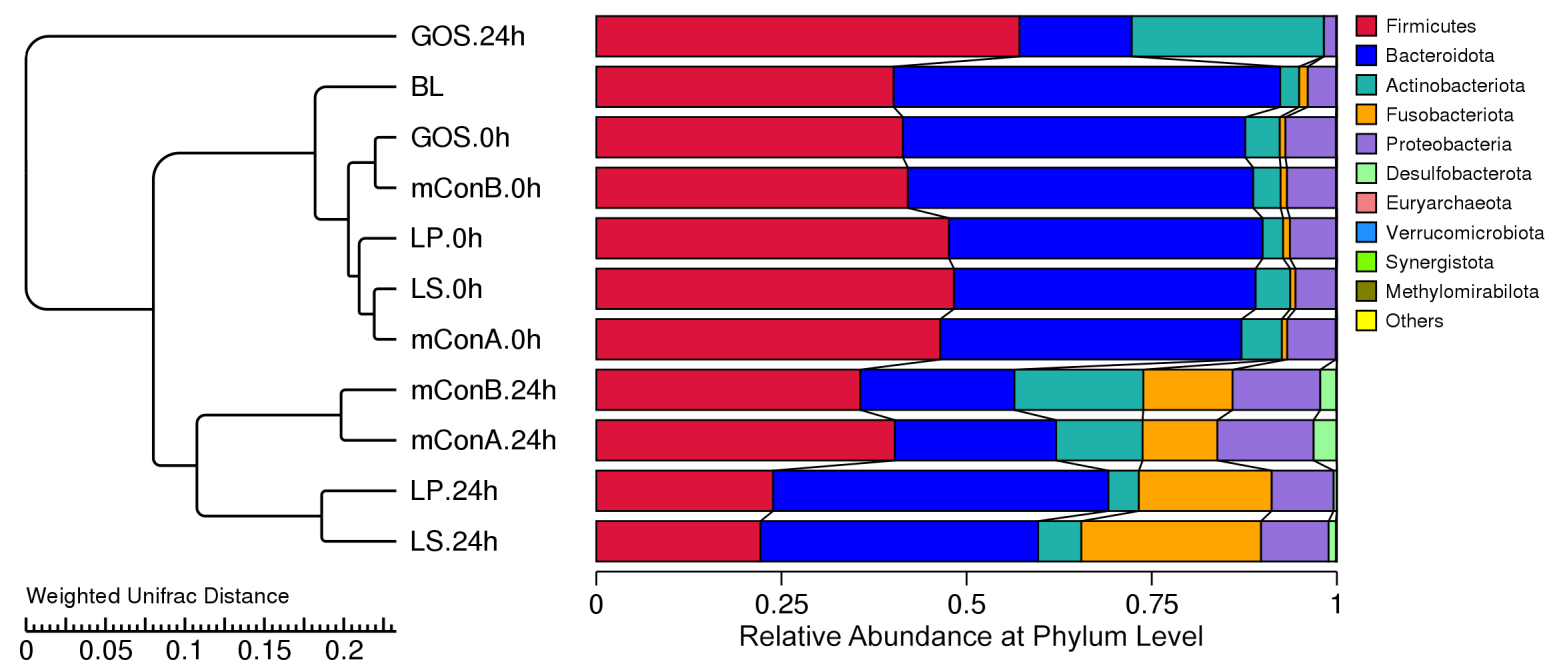
UPGMA cluster tree based on weighted UniFrac distance shows evolutionary relationships between different treatments in dataset. UPGMA tree (left) and relative abundance in phylum map (right) are depicted. Treatments are grouped based on their similarities in composition and structure.

Principal component analysis (Fig. 10A) showed that the treatments could be divided into three main groups: group 1 included the treatments LP.24 h and LS.24 h; group 2 included the treatments BL, LP.0 h, LS.0 h, GOS.0 h, mConA.0 h, and mConB.0 h; and group 3 included the treatments mConA.24 h and mConB.24 h. The GOS.24 h treatment group was clearly different from the other groups but was most similar to group 1. This was similar to the principal coordinate analysis based on the weighted UniFrac distance, which also demonstrated a clear distinction between the treatments (Fig. 10B). Based on the unweighted pair-group method with arithmetic mean, which was used to generate a cluster tree of the treatment groups, three major groups were distinguished based on species composition and structure (Fig. 11). Thus, there were distinct differences in the microbial community composition between treatments, suggesting that crude polysaccharide (LP and LS) treatments notably affected the gut microbiota.

### Effect of crude polysaccharides LP and LS on fecal gut bacterial community at different taxonomic levels

**Figure 12:**
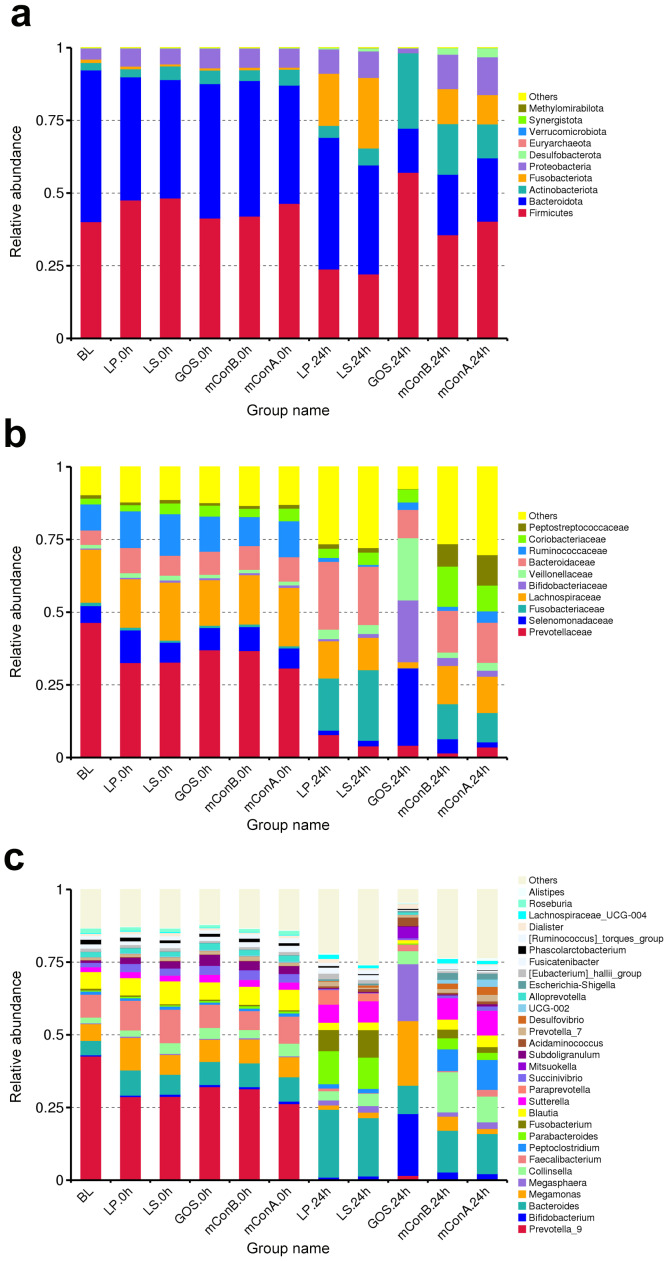
Bacterial profiling at different taxonomic levels of treatments. (A) Phylum. (B) Family. (C) Genus. *Y*-axis represents “Relative Abundance,” and *X*-axis represents “Group Name.”. In Figures (A) and (B), “Others” represents the total relative abundance of the rest of the phyla besides the top 10 phyla or families.

The impact of the crude polysaccharides LS and LP on the fecal gut bacterial community at three taxonomic levels (phylum, family, and genus) is described in this study. At the phylum level (Fig. 12A), the top five bacterial phyla, Firmicutes, Bacteroidota, Actinobacteriota, Fusobacteriota, and Proteobacteria were found in all treatments. A comparison of bacterial growth at 0 and 24 h of fermentation showed that the crude polysaccharide LP greatly increased the growth of the bacterial groups Actinobacteriota, Fusobacteriota, Proteobacteria, and Desulfobacteriota (*p* < 0.05), but significantly decreased the number of bacteria in the Firmicutes group (*p* < 0.01). These results are consistent with those of fecal fermentation with LS. The phylum Bacteroidota was slightly reduced after fermentation with LP, whereas the opposite was observed with LS. Although the reference prebiotic GOS strongly promoted the growth of bacteria in the phyla Firmicutes and Actinobacteria, a reduction in the abundance of Fusobacteriota, Proteobacteria, and Desulfobacteriota was observed. Unfortunately, a statistical analysis could not be performed because of the small sample size of the GOS group. Moreover, it was revealed that, in the control groups, both mConA (the use of sterile distilled water instead of crude polysaccharides) and mConB (the solid residue and the supernatant of the control tube in the simulated gastrointestinal tract digestion) also significantly increased the growth of bacteria in the phyla Fusobacteriota and Proteobacteria (*p* < 0.05). Relative abundances at the phylum level are listed in Table S7.

At the family level (Fig. 12B), both crude polysaccharides LS and LP promoted the growth of bacteria in similar family taxa. The major bacterial families found in all the treatments were Prevotellaceae, Selenomonadaceae, Fusobacteriaceae, Lachnospiraceae, Bifidobacteriaceae, Veillonellaceae, Bacteroidaceae, Ruminococcaceae, Coriobacteriaceae, and Peptostreptococcaceae. Compared with the beginning of fermentation (0 h), both LS.24 h and LP.24 h showed a notable increase (*p* < 0.05) in the number of bacteria belonging to the families Bacteroidaceae, Fusobacteriaceae, Tannerellaceae, Sutterellaceae, Veillonellaceae, and Bifidobacteriaceae. In contrast, a significant decrease (*p* < 0.05) was observed in Prevotellaceae, Selenomonadaceae, Lachnospiraceae, and Ruminococcaceae. The GOS.24 h sample group exhibited a notable increase in bacteria from Selenomonadaceae, Bifidobacteriaceae, Veillonellaceae, and Bacteroidaceae families. However, there was a decrease in the abundance of Prevotellaceae, Fusobacteriaceae, and Lachnospiraceae. Both control groups exhibited comparable microbial successions after 24 h of fermentation. In mConA, a significant reduction in Prevotellaceae (*p* < 0.001) was accompanied by the expansion of Fusobacteriaceae (*p* = 0.001) and Peptostreptococcaceae (*p* < 0.001). Similarly, mConB displayed a significant decline in Prevotellaceae (*p* = 0.001) and concurrent increases in Fusobacteriaceae (*p* = 0.001), Peptostreptococcaceae (*p* = 0.003), and Coriobacteriaceae (*p* = 0.025). The relative abundances at the family level are listed in Table S8.

Figure 12C exhibits the variations in bacterial genera of the different treatments at 0 and 24 h after *in vitro* fermentation. The LP treatment of 24-h fermentation (LP.24h) stimulated the growth of bacterial genera *Bacteroides*, *Parabacteroides*, *Fusobacterium*, *Paraprevotella*, and *Sutterella*, which was significantly higher (*p* < 0.01) than that of the LP.0 h group. However, the abundance of the genera *Prevotella*, *Megamonas*, *Faecalibacterium*, and *Blautia* was significantly decreased (*p* < 0.05). These results were similar to those of the LS treatment group. Comparing variation in bacterial genera within the GOS treatment group, it was demonstrated that the genera *Bifidobacterium*, *Megamonas*, and *Megasphaera* were strongly increased after 24 h of fermentation (GOS.24h), while *Prevotella* _9 was clearly reduced. In the control groups, after 24 h of fermentation, mConA exhibited significant reductions (*p* < 0.05) in the relative abundances of *Prevotella*, *Megamonas*, *Faecalibacterium*, and *Blautia*. Conversely, significant increased (*p* < 0.05) were observed for *Peptoclostridium*, *Parabacteroides*, and *Fusobacterium*. This community profile at the genus level was similar to the microbial community shifts observed in the mConB control group. The relative abundance at the genus taxonomic level is shown in Table S9.

### Crude polysaccharides reduced Firmicutes/Bacteroidota and Blautia/Bacteroides ratios

**Figure 13:**
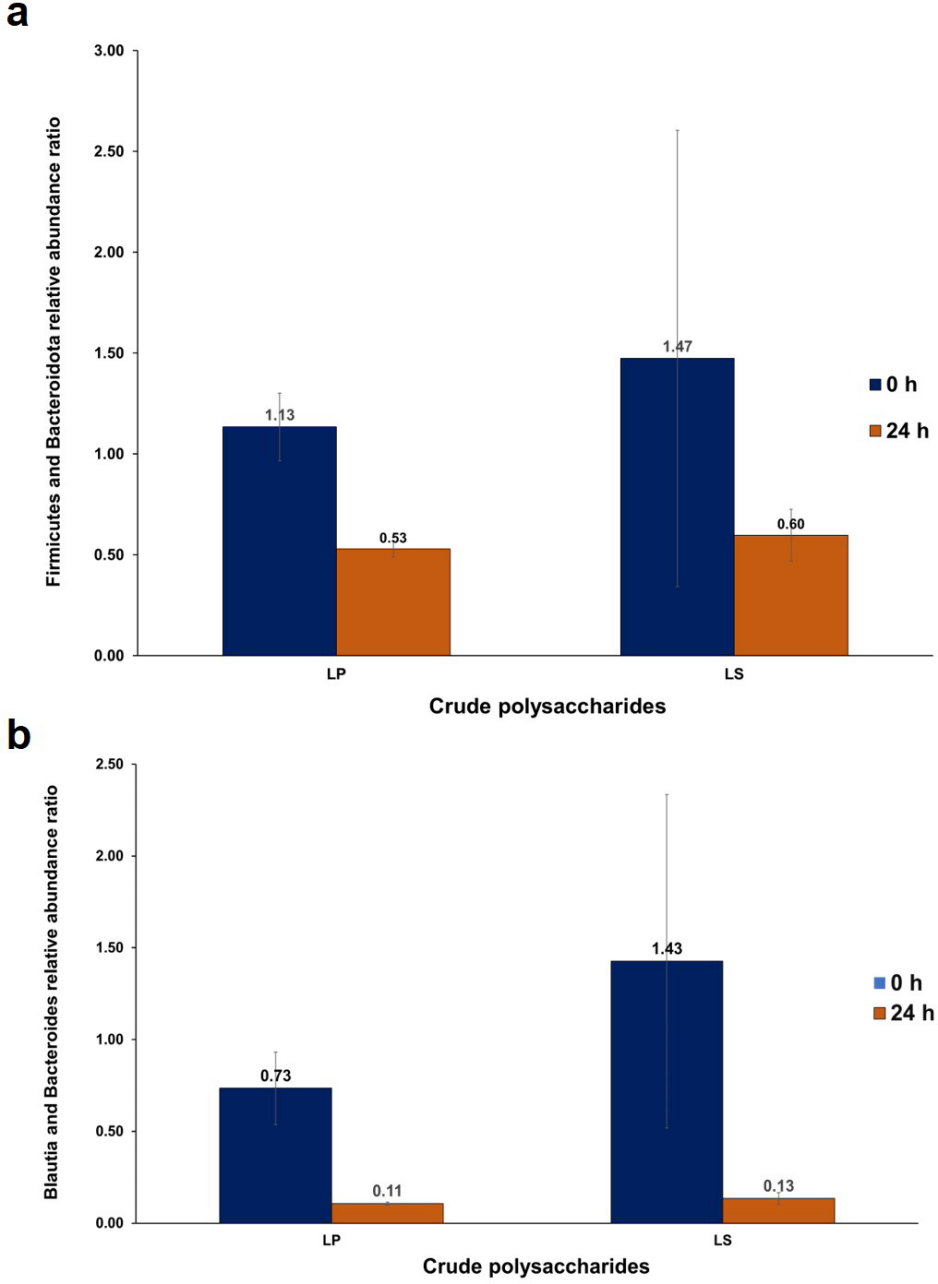
Firmicutes/Bacteroidota and Blautia/Bacteroides ratio. Firmicutes/Bacteroidota ratio (A) and Blautia/Bacteroides ratio (B) under LS and LP treatments at 0 h and 24 h. The *x*-axis represents the type of crude polysaccharide (LS or LP), and the *y*-axis represents the corresponding ratio values. Comparisons of the Firmicutes/Bacteroidota and Blautia/Bacteroides relative abundance ratios between 0 h and 24 h for each treatment were performed using the Wilcoxon signed-rank test.

At the phylum level, fermentation reduced the relative abundance of Firmicutes in both LP and LS treatment groups (*p* < 0.001 for LP; *p* < 0.05 for LS), whereas Bacteroidota showed a modest but non-significant increase. Accordingly, the Firmicutes/Bacteroidota ratio decreased in both treatments (Fig. 13A and Table S10). For the LS group, comparisons of the Firmicutes/Bacteroidota ratio between 0 and 24 h were further evaluated using the Wilcoxon signed-rank test because of the small sample size and non-normal paired distribution. The median ratio decreased from 0 h (0.9408) to 24 h (0.5839); although this reduction was not statistically significant (*p* = 0.125), all biological replicates demonstrated a consistent downward trend after LS exposure.

At the genus level, the Blautia/Bacteroides ratio decreased after 24 h of fermentation driven by a significant increase in *Bacteroides* (*p* = 0.003 for LP; *p* < 0.001 for LS) and a significant reduction in *Blautia* (*p* < 0.001 for LP; *p* = 0.005 for LS), resulting in lower ratios at 24 h than at 0 h (Fig. 13B and Table S10). In the LP group, the Blautia/Bacteroides ratio was further evaluated using the Wilcoxon signed-rank test owing to the small sample size. The median ratio decreased from 0.7492 at 0 h to 0.1040 at 24 h; however, the difference was not statistically significant (*p* = 0.1250), indicating a modest shift in the community structure toward increased *Bacteroides* dominance, although the change was not statistically significant.

### Change in pH after fermentation

**Figure 14:**
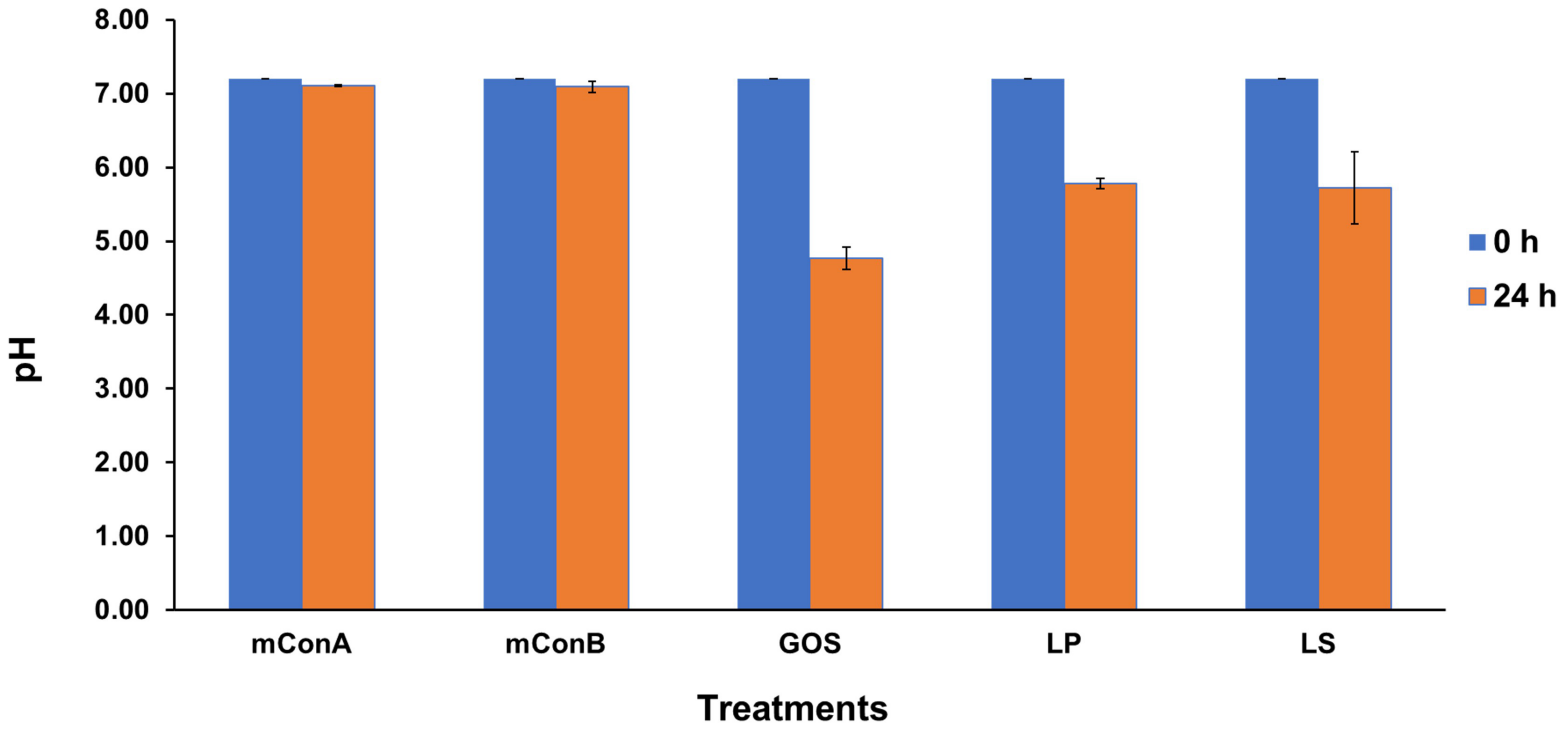
Change in pH in each treatment group. Change in pH in each treatment group (mConA, mConB, GOS, LP, and LS) at 0 and 24 h after fermentation. Paired comparisons between 0 h and 24 h for each treatment were performed using the Wilcoxon signed-rank test.

As shown in Fig. 14 and Table S11, the initial pH of all treatments was approximately 7.20. The treatment groups LS.24 h and LP.24 h showed a reduction in the pH. The average pH values in the LS.24 h and LP.24 h groups were 5.73 and 5.78, respectively. The GOS.24 h treatment group had an average pH value of 4.77, whereas the control groups mConA and mConB showed a slight decrease in pH, with average pH values of 7.11 and 7.09, respectively. Paired comparisons of pH between 0 and 24 h for each treatment (mConA, mConB, GOS, LP, and LS) using the Wilcoxon signed-rank test showed no statistically significant changes (*p* > 0.05), although a decreasing trend in pH was observed, particularly in the GOS, LP, and LS groups.

### Deposition of sequencing data in public databases

The LSU-28S rRNA gene sequences of *L. polychrous* LP1 and *L. squarrosulus* PB1 were deposited in the NCBI database under the accession numbers PQ340139 and OR554123, respectively. In addition, the 16S rRNA gene sequencing data generated in this study were deposited in the European Nucleotide Archive at EMBL-EBI under project accession number PRJEB110864 (https://www.ebi.ac.uk/ena/browser/view/PRJEB110864).

## Discussion

Recently, polysaccharides extracted from edible mushrooms, such as *P. eryngii*, *T. fuciformis*, *A. bisporus*, and *H. erinaceus*, have been found to positively influence gut microbial composition (Christodoulou et al., 2023; Duan et al., 2023; Tian et al., 2022; Zhu et al., 2024). This was the basis of our study, in which two mushroom mycelial isolates, PB1 and LP1, were collected from wild mushroom mycelia. These two isolates were initially identified as belonging to the genus *Lentinus* spp. based on their fruiting body appearance and mycelial morphology. However, this species has not been identified yet. Thus, molecular identification techniques offer a more accurate and reliable approach for identifying mushrooms, addressing the shortcomings of traditional methods, and enhancing the safety and quality of mushroom consumption and commerce (Raja et al., 2017). Based on PCR amplification of the 28S rRNA gene, followed by nucleotide sequencing and analysis, the mushroom isolates PB1 and LP1 were identified as *L. squarrosulus* and *L. polychrous*, respectively. This result is consistent with that of the phylogenetic analysis. Thus, strains PB1 and LP1 were designated *L. squarrosulus* PB1 and *L. polychrous* LP1, respectively.

For future downstream applications, sufficient quantities of mushrooms (fruiting bodies) must be produced. Compared with cultivated mushrooms, it has been reported that wild edible mushrooms have a higher quantity of nutritional content, flavorants, and medicine-associated compounds (Mleczek et al., 2021), but the consistency of their quality depends on their exposed environment, leading to the uncontrollable consistency of cultivation. Therefore, cultivation of mushrooms in more controllable systems, such as greenhouses, is an alternative strategy to ensure consistent quality and sufficient mushroom yield. Our study demonstrated the use of a plastic bag-based method for greenhouse cultivation of *L. squarrosulus* PB1 and *L. polychrous* LP1. This method requires minimal equipment and space and can be performed indoors without any specialized climate control, thus increasing the possibility of increasing mushroom yield. Our results demonstrated that mushrooms could be harvested within two weeks of inoculation. This method has been successfully applied to cultivate medicinal mushrooms, such as *Lentinula edodes* (Shiitake), *Ganoderma lucidum* (Reishi), and *Auricularia polytricha* (Jeewanthi, Ratnayake & Rajapakse, 2017; Lee et al., 2012; Wu, Liang & Liang, 2020). However, a comparison of the nutritional value and other compounds present in both wild and cultivated mushrooms was not performed in this study.

Numerous studies have explored the bioactive chemicals found in edible mushrooms that exhibit a range of health-promoting properties, such as anticancer, antiviral, and antioxidant activities, and alleviate the severity of inflammatory bowel disease by modifying the gut microbiota (Baruah et al., 2023; Deveci et al., 2024; Ma et al., 2021; Zhao et al., 2023b). Polysaccharides from edible mushrooms can be extracted using various methods, among which hot water extraction (HWE) is the most common method because of its simplicity and effectiveness (Cheng et al., 2024). Studies have demonstrated that optimizing HWE conditions can enhance the yield of polysaccharide extracts, particularly by increasing agitation, temperature, and fermentation period (Khalil & Lukasiewicz, 2024; Sangthong et al., 2022). Our results indicated that incorporating sonication at the initial stage of the HWE method could enhance the extraction yields of crude polysaccharides from *L. squarrosulus* PB1 and *L. polychrous* LP1, which were slightly higher than those reported in our previous study and higher than those of other mushroom species, including *Grifola frondose* and *Inonotus obliquus* (Hwang et al., 2019; Panya et al., 2024; Su, Lai & Ng, 2017). This enhancement may be due to sonication, which disrupts the fungal cell wall structure and facilitates the subsequent release of intracellular polysaccharides during HWE. Our results showed that the crude polysaccharides from *L. squarrosulus* and *L. polychrous* contained notably higher phenolic compound concentrations than those reported previously (*L. squarrosulus*, 1.52 ± 0.01 mg/100 g; *L. polychrous*, 0.91 ± 0.03 mg/100 g) (Srikram & Supapvanich, 2016). Thus, cultivated mushrooms, including *L. squarrosulus* and *L. polychrous*, are good sources of polysaccharides and phenolic compounds, which can be further evaluated for their bioactive functions. Phenolic compounds have been reported to modulate gut bacterial communities, suggesting that they may have contributed to the microbiome changes observed in this study (Cardona et al., 2013; Selma, Espín & Tomás-Barberán, 2009). In our ongoing study, we are purifying these crude polysaccharide extracts to clarify their specific roles and independent effects on the modulation of bacterial communities.

There is an exponentially growing interest in evaluating the effects of mushroom-derived polysaccharides on the human gut microbiome, particularly their ability to modulate the gut bacterial community and enhance health through various mechanisms. Additionally, the initial testing of the fermentation of polysaccharide extracts with microorganisms in human feces involved subjecting the extracts to simulated human gastrointestinal conditions, including those in the oral cavity, stomach, and intestine. This simulation realistically mimics food intake in the body. Previous studies tested simulated gastrointestinal digestion prior to fermentation using polysaccharide extracts from *Helicteres angustifolia* L., *Volvariella volvacea*, and *Oudemansiella radicata* (Chen et al., 2019; Hu et al., 2023; Liu et al., 2021). These studies have shown that the upper gastrointestinal conditions in humans did not affect the structure of polysaccharide extracts from mushrooms, indicating the potential prebiotic properties of these polysaccharides. This method was used in this study. The crude polysaccharides LP and LS exhibited high resistance to simulated gastrointestinal digestion, indicating that they may reach the colon intact and be available for microbial fermentation. This digestion-resistant property aligns with reports that mushroom polysaccharides, including β-glucans, maintain structural stability throughout the gastrointestinal tract and support beneficial microbial growth (Hu et al., 2023; Ye et al., 2025). These findings highlight the potential of *Lentinus*-derived polysaccharides as promising prebiotic candidates.

In this study, four fecal samples from four individuals were included in the *in vitro* fermentation process. Although these sample sizes may be sufficient for statistical analysis, using a larger and more diverse cohort of donors would better represent the inter-individual variability of the human gut microbiota. Based on this study, the results of the 16S rRNA gene amplicon sequencing of the samples revealed that the number of effective reads had an average length of 417 nucleotides, which was reflected in the length of the nucleotide sequence in the V3–V4 region (400–500 nucleotides) of general bacteria (Yarza et al., 2014). Thus, these sequencing outputs had acceptable quality for further data analysis and interpretation. According to principal performance, the short-read sequencing technique is reliable and accurate at the lowest taxonomy at the genus level (Gehrig et al., 2022); thus, taxonomic profiling of the phylum, family, and genus is described in this study. After *in vitro* batch fermentation, the crude polysaccharides LP and LS led to a significant shift in the gut bacterial community. The rank abundance curves and alpha diversity indices indicated a reduction in bacterial diversity, especially in the species evenness value, indicating that the polysaccharides LP and LS specifically supported the growth of some dominant bacterial species. These findings aligned with previous research suggesting that dietary polysaccharides can influence microbiome composition by supporting specific bacterial growth while suppressing certain taxa (Álvarez-Mercado & Plaza-Diaz, 2022).

Analysis of the bacterial community-modulating properties of the LS and LP crude polysaccharides at the phylum level revealed that both crude polysaccharides LS and LP supported the growth of similar bacterial phyla. Of these, Firmicutes, Bacteroidota, Actinobacteriota, Fusobacteriota, and Proteobacteria were predominant, which is consistent with their well-known dominance in the human gut microbiota (Almeida et al., 2019). These bacterial groups possess diverse carbohydrate-active enzymes that enable the degradation of complex polysaccharides, particularly fucose- and mannose-rich substrates (Flint et al., 2012). As both LS and LP polysaccharides share similar sugar compositions, with fucose as the major component (Panya et al., 2024), their characteristic sugar compositions likely favored the selective growth of these dominant bacterial phyla during fermentation. It has been reported that monosaccharides present in a polysaccharide chain are one of the polysaccharide characteristics that affect the regulatory function of gut microbiota (Zhao et al., 2023a). A heteropolysaccharide, a polysaccharide composed of different types of monosaccharides (mainly glucose, fucose, galactose, and mannose), usually has a greater effect on gut microbiota modulation than a mushroom homopolysaccharide such as β-glucan (Luo et al., 2018). However, the relationship between the structures of mushroom polysaccharides, including crude polysaccharides LS and LP, and the gut microbiota remains unclear. Further studies on the interaction between mushroom polysaccharides and the gut microbiota are an attractive research topic.

In the absence of fermentable carbohydrates, the enrichment of Proteobacteria and Fusobacteriota observed in mConA and mConB likely reflects a metabolic shift toward taxa capable of proteolytic fermentation or the utilization of endogenous nitrogenous substrates present in the basal medium, such as peptone or yeast extract. Under these substrate-limited conditions, metabolically flexible and opportunistic Proteobacteria may rapidly proliferate, whereas genera specialized in complex polysaccharide degradation, including *Prevotella*, *Faecalibacterium*, and *Blautia*, exhibit marked declines consistent with nutrient limitation. Similar patterns have been reported in *in vitro* fermentation studies showing that the absence of fermentable substrates leads to reduced microbial diversity and a relative enrichment of taxa capable of protein utilization or survival under low-energy conditions (Gu et al., 2021; Yuan et al., 2025). The overall similarity between mConA and mConB further indicates that the inclusion of simulated digestive fluids does not substantially alter baseline microbial community structure, supporting the use of INFOGEST digestion conditions as a physiologically relevant background for evaluating substrate-specific effects (Minekus et al., 2014).

Compared with the other treatment groups after fermentation, the phyla Bacteroidota and Fusobacteriota were relatively more abundant in the LP and LS treatment groups. This taxonomic profile was similar at the family and genus levels, and the bacterial members of these two phyla were predominant. This is consistent with previous reports that polysaccharides from the mushrooms *Volvariella volvacea* and *Boletus auripes* promote the growth of *Bacteriodes* (Hu et al., 2023; Luo et al., 2023). Members of the phylum Bacteroidota (formerly Bacteroidetes) are well-recognized keystone degraders in the human gut microbiota and are equipped with diverse carbohydrate-active enzymes and polysaccharide utilization loci that enable them to break down complex glycans (Shin et al., 2024). These enzyme groups allow *Bacteroides* to selectively recognize and metabolize fiber-derived glycans, thus influencing both interspecies competition and microbial network dynamics in the gut ecosystem, as demonstrated by Patnode et al. (2019). Many Bacteroidota species, such as *Bacteroides thetaiotaomicron* and *Bacteroides uniformis,* are the primary bacteria that break down mushroom carbohydrates in the human gut (Qu et al., 2025; Shi et al., 2024). This may explain the predominance of these bacteria in LP and LS treatment groups.

In addition to *Bacteroides*, *Parabacteroides*, a member of the phylum Bacteroidota, also increased in abundance after fermentation with crude polysaccharides LS and LP. Recent studies have demonstrated that *Parabacteroides* species are the predominant bacteria in fermented mushroom-derived polysaccharides, such as *Tremella fuciformis*, *Flammulina velutipes*, and *Wolfiporia cocos* (Fang et al., 2024; Ma et al., 2023; Wang et al., 2024). Certain species within the genus *Parabacteroides*, particularly *Parabacteroides distasonis* and *Parabacteroides goldsteinii*, have demonstrated probiotic potential. Recent studies have demonstrated that *Parabacteroides distasonis* exerts beneficial effects in various disease models by modulating host immunity and metabolism, alleviating inflammatory arthritis, reducing colitis severity through immune and microbiota regulation, and improving obesity-related metabolic dysfunction through succinate and secondary bile acid production (Kverka et al., 2011; Sun et al., 2023; Wang et al., 2019). These findings support its potential as a next-generation probiotic, as these candidates are often commensal microbes with clearly defined mechanisms, such as producing bioactive metabolites or influencing host signaling pathways, traits that *P. distasonis* has already been shown to possess (O’Toole, Marchesi & Hill, 2017).

As expected, the number of beneficial bacteria, especially *Bifidobacterium* species, was significantly higher in the GOS treatment group than in the other treatment groups. This is reasonable because the prebiotic properties of GOS selectively support bifidobacterial growth (Li et al., 2015). Thus, GOS is often included in synbiotic formulations. As mentioned previously, the phylum Fusobacteriota also increased in the LP and LS treatment groups. This increase may be attributed to substrate specificity. It has been reported that the degree of polymerization (DP) in the polysaccharide chain affects human gut microbial diversity and abundance (Chen et al., 2020). The *in vitro* fecal fermentation of three substances that differ in DP, carboxymethylcellulose (the highest DP), β-glucans (an intermediate level of DP), and galactooligosaccharides (the lowest DP), showed that carboxymethylcellulose and β-glucans promoted an increase in *Fusobacterium* and *Bacteroides* species. This indicates that high-DP polysaccharides support the proliferation of *Fusobacterium* species (Chen et al., 2020). The effects of the DP of the crude LS and LP polysaccharides on the gut microbiome were not investigated in this study. Thus, a study on the effects of the DP of polysaccharide-rich substances, including LP and LS, on the modulation of the human gut microbiota is currently being planned.

In addition to the increase in the abundance of Bacteroidota, the abundance of Firmicutes was reduced in the LP and LS treatment groups compared with the other groups. This phylum consists of bacteria that are recognized as beneficial bacteria, such as the family of Lactobacillaceae, and as harmful bacteria, such as the family of Clostridiaceae. The Firmicutes/Bacteroidota and Blautia/Bacteroides ratios were used as indicators of microbiota balance. It has been reported that the Firmicutes/Bacteroidota and Blautia/Bacteroides ratios in the gut were positively correlated to specific health conditions, and high Firmicutes/Bacteroidota and Blautia/Bacteroides ratios have been directly associated with obesity in patients (Kim et al., 2022; Woting & Blaut, 2016). Our results indicated a downward trend in the Firmicutes/Bacteroidota ratio in both the LS and LP treatment groups, although this change did not reach statistical significance. At the genus level, the Blautia/Bacteroides ratio also decreased after fermentation, reflecting a shift toward increased *Bacteroides* and reduced *Blautia*, although the overall change in the ratio was modest and not statistically significant. There have been reports that individuals with obesity and type 2 diabetes have a significantly higher Firmicutes/Bacteroidota ratio compared with normal weight individuals (Ahmed et al., 2024). Conversely, some studies have reported that the Firmicutes/Bacteroidota ratio does not differ between individuals with type 2 diabetes and those of normal weight (Magne et al., 2020; Muheyati et al., 2024), suggesting that other factors beyond the Firmicutes/Bacteroidota ratio may be involved in obesity and diabetes, and the Firmicutes/Bacteroidota ratio alone may not be a definitive biomarker for obesity. This finding is similar to the association between the Blautia/Bacteroides ratio and obesity. Therefore, further research is needed to decipher the roles of Firmicutes/Bacteroidota and Blautia/Bacteroides ratios in specific health conditions, including obesity and type 2 diabetes mellitus.

The pH tended to decrease in the GOS, LS, and LP treatment groups at 24 h compared with that at 0 h; however, these changes were not statistically significant. Changes in pH may be reflected in the metabolomic products produced during the fermentation process, such as SCFAs and other organic compounds. Recently, *in vitro* fecal fermentation of polysaccharides derived from the mushroom *Poria cocos* resulted in a reduction in pH and an increase in SCFA production, particularly acetic and propionic acids. These changes indicated that *P. cocos* polysaccharides were effectively metabolized by the gut microbiota (Zhou et al., 2025). However, the metabolomic products of fermentation were not determined in this study. Thus, the determination of metabolomic end products in the fermentation medium will be performed in future investigations.

## Conclusions

The species *L. squarrosulus* PB1 and *L. polychrous* LP1 were accurately identified using molecular techniques. Crude polysaccharides were extracted, characterized, and used as substrates for *in vitro* batch fermentation of fecal microbiota samples. These crude polysaccharides preferentially enhanced the population of bacteria in the phylum Bacteroidota, especially the genera *Bacteroides* spp. and *Parabacteroides* spp. The phylum Fusobacteriota, a potentially harmful bacterium, also increased after fermentation with the two polysaccharides. However, further investigations are required to determine their functional significance under specific health conditions. This study had some limitations. First, although cultivation conditions and morphological uniformity of the mushrooms were controlled prior to extraction, the lack of proximate and physicochemical analyses limits verification of raw mushroom batch consistency. Second, the use of fecal samples from a small number of donors of a single sex may not adequately represent the inter-individual variability of the human gut microbiota, thus limiting the generalizability of the results. Third, the *in vitro* fermentation model may not fully replicate *in vivo* conditions, particularly host immune responses and complex physiological interactions. Additionally, the current experimental design did not include a specific control for digestive enzymes; thus, the potential influence of residual enzymes as substrates for the microbiota could not be entirely ruled out. Finally, this study focused on bacterial composition and did not include metabolomic profiling, such as SCFA quantification, which would provide additional insights into the functional consequences of the observed bacterial community shifts. A metabolomic analysis is currently being performed in our ongoing research to complement these findings.

##  Supplemental Information

10.7717/peerj.21348/supp-1 Supplemental Information 1 Dataset used to calculate the composition of extracted crude polysaccharides

10.7717/peerj.21348/supp-2 Supplemental Information 2 Dataset used to calculate the resistance of crude polysaccharides to simulated human gastrointestinal digestion

10.7717/peerj.21348/supp-3 Supplemental Information 3 Quantity and purity of genomic DNA extracted from fermentation samples

10.7717/peerj.21348/supp-4 Supplemental Information 4 Nucleotide sequencing quality for each sample

10.7717/peerj.21348/supp-5 Supplemental Information 5 the Shannon diversity values for treatments

10.7717/peerj.21348/supp-6 Supplemental Information 6 Alpha diversity indices and Kruskal–Wallis test results for each treatment group

10.7717/peerj.21348/supp-7 Supplemental Information 7 Relative abundance of bacterial phyla across treatments

10.7717/peerj.21348/supp-8 Supplemental Information 8 Relative abundance of bacterial families across treatments

10.7717/peerj.21348/supp-9 Supplemental Information 9 Relative abundance of bacterial genera across treatments

10.7717/peerj.21348/supp-10 Supplemental Information 10 Calculation of Firmicutes/Bacteroidota and Blautia/Bacteroides relative abundance ratios

10.7717/peerj.21348/supp-11 Supplemental Information 11 pH values before and after fermentation in each treatment group

10.7717/peerj.21348/supp-12 Supplemental Information 12 Sample groups and sampling time points used in the *in vitro* fermentation and subsequent genomic DNA extraction The chart summarizes the number of samples collected from each treatment group (BL, mConA, mConB, GOS, LP, and LS) at 0 h and 24 h during the in vitro fermentation experiment. A total of 21 samples were obtained at 0 h and 18 samples at 24 h for downstream genomic DNA extraction and sequencing.

10.7717/peerj.21348/supp-13 Supplemental Information 13 Ethidium bromide-stained gel showing amplified product of partial 16S ribosomal RNA gene (16S rRNA) of bacteria of samples collected at 0 h (a) and 24 h (b) after fermentation Lane M contained VC DNA ladder. Lane P contained PCR product with genomic DNA of *Escherichia coli* as a positive control. Lane N contained PCR product without any DNA template. Amplified products of different samples were explained as textually embedded in figure. Arrow indicates amplified product size in base pairs (bp.).

## Additional Information and Declarations

### Data Availability

The following information was supplied regarding data availability: The LSU-28S rRNA gene sequences of L. polychrous LP1 and L. squarrosulus PB1 are available at NCBI: PQ340139 and OR554123, respectively. In addition, the 16S rRNA gene sequencing data generated in this study are available at the European Nucleotide Archive at EMBL-EBI: PRJEB110864.
