# Supplementary figures and images for: In vitro modulation of the human gut bacterial community by crude polysaccharides from cultivated mushrooms Lentinus polychrous and Lentinus squarrosulus
Source: PeerJ. 2026 Jun 10;14:e21348. doi: 10.7717/peerj.21348 (PMC13264279; doi:10.7717/peerj.21348)

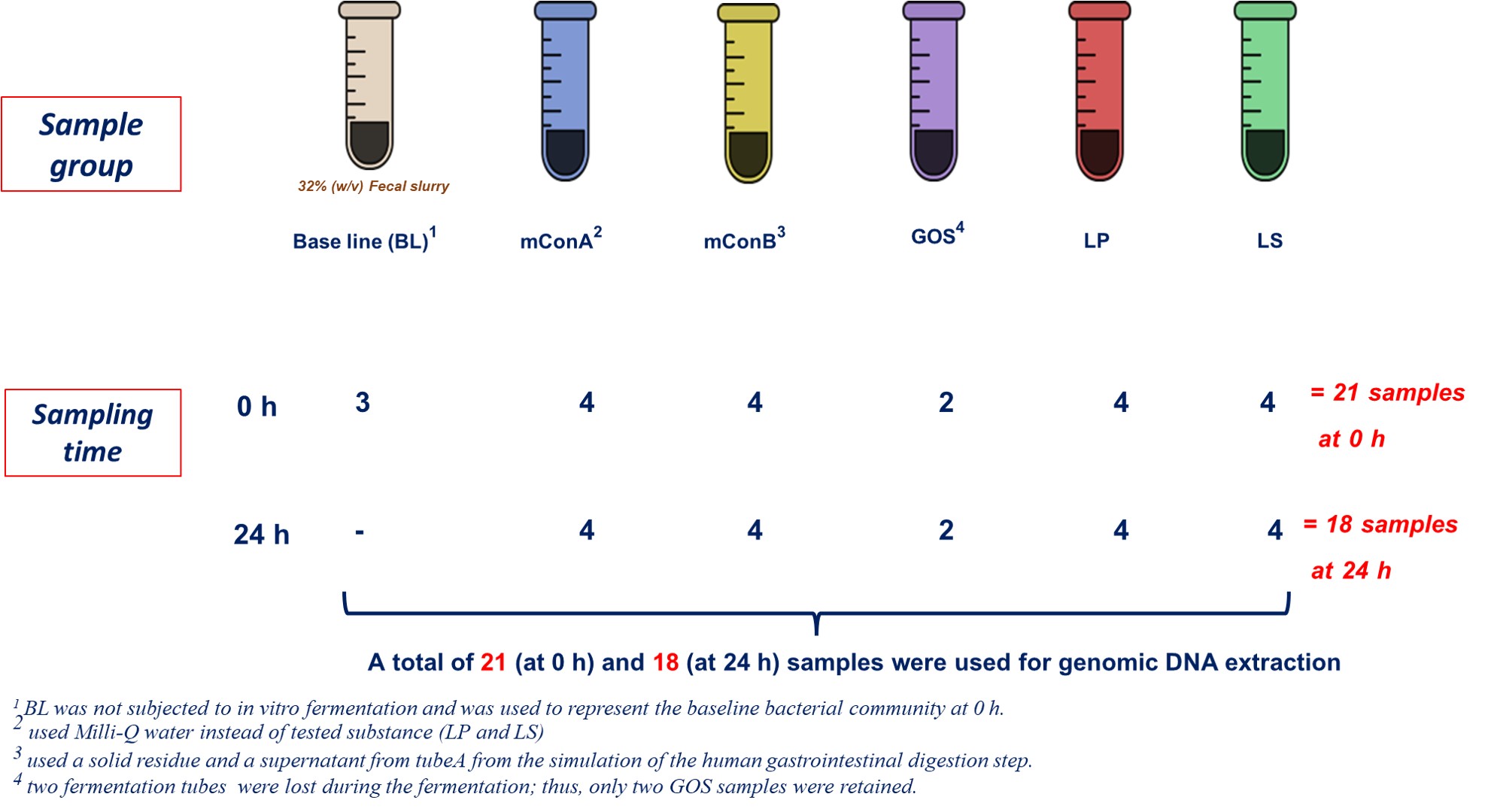

Supplement: Supplemental Information 12 — The chart summarizes the number of samples collected from each treatment group (BL, mConA, mConB, GOS, LP, and LS) at 0 h and 24 h during the in vitro fermentation experiment. A total of 21 samples were obtained at 0 h and 18 samples at 24 h for downstream genomic DNA extraction and sequencing. [file peerj-14-21348-s012.jpg]

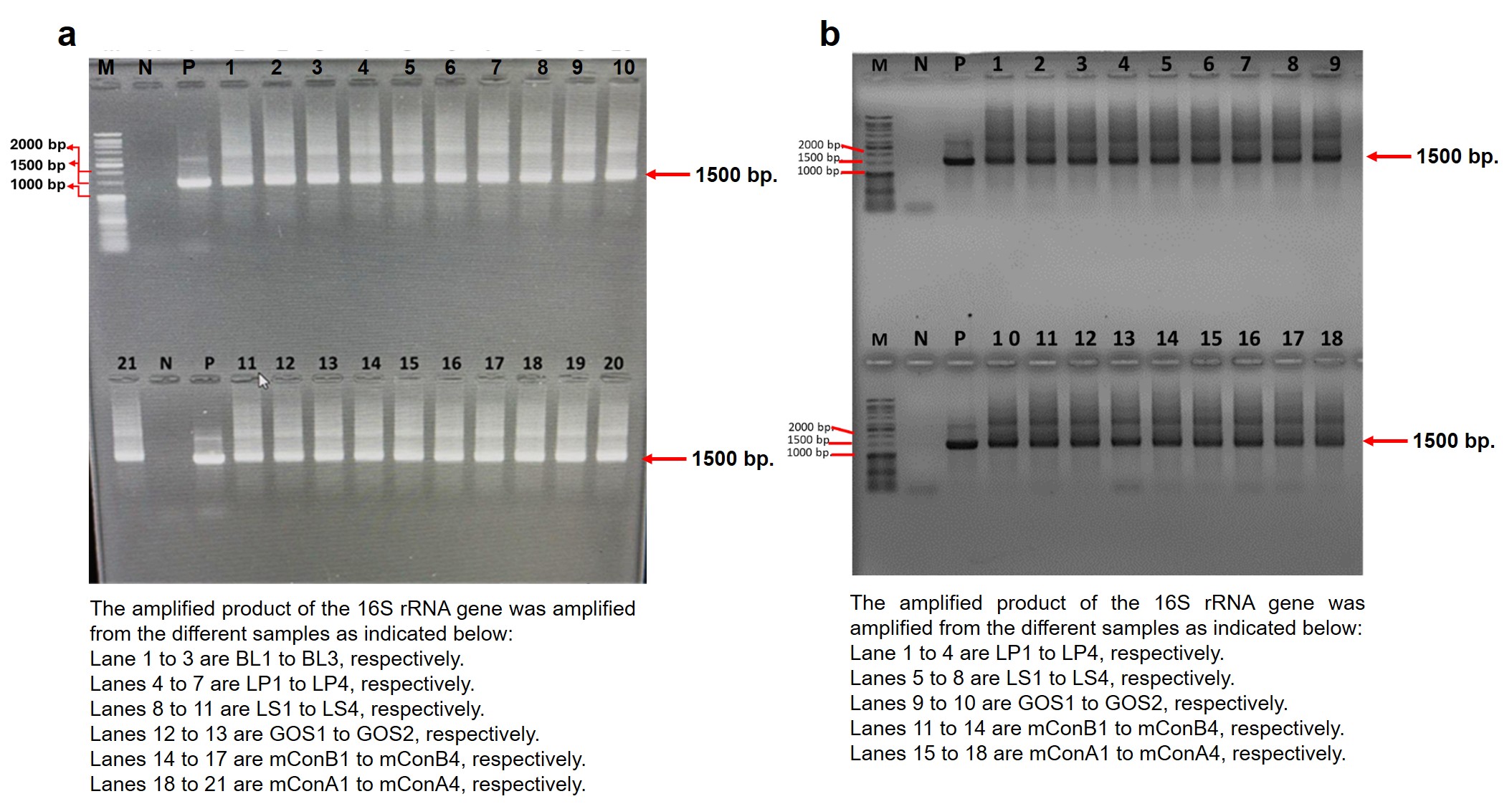

Supplement: Supplemental Information 13 — Lane M contained VC DNA ladder. Lane P contained PCR product with genomic DNA of Escherichia coli as a positive control. Lane N contained PCR product without any DNA template. Amplified products of different samples were explained as textually embedded in figure. Arrow indicates amplified product size in base pairs (bp.). [file peerj-14-21348-s013.jpg]
